# Standard precautions versus clinically triggered contact precautions for prevention of extended-spectrum β-lactamase-producing enterobacterales in acute-care geriatric units: A prospective non-inferiority interventional study protocol (GERSP-study)

**DOI:** 10.1371/journal.pone.0353783

**Published:** 2026-07-21

**Authors:** Olfa Ezzi, Laurie Renaudin, Christophe Goetz, Arpiné Ardzivian Elnar, Azzeddine Azzemou, Noel Blettner, Mathieu Llorens

**Affiliations:** 1 Infection Control Department, Regional Hospital Centre Metz-Thionville, Metz, France; 2 Clinical Research Support Unit, Regional Hospital Centre Metz-Thionville, Metz, France; 3 Geriatric Department, Regional Hospital Centre Metz-Thionville, Metz, France; Universidad San Francisco de Quito, ECUADOR

## Abstract

**Introduction:**

Extended-spectrum β-lactamase–producing Enterobacterales (ESBL-E) pose a major global public health challenge, as they are associated with frequent empirical antibiotic treatment failure and increased patient morbidity and mortality. In this context, contact precautions (CP) have traditionally been recommended for hospitalized patients colonized or infected with ESBL-E to prevent healthcare-associated transmission. However, growing evidence has questioned the effectiveness of this strategy in certain care settings.

The primary objective of this study is to assess the non-inferiority of standard precautions (SP) compared with **clinically triggered** CP in reducing the incidence density of ESBL-E acquisition in acute-care geriatric wards. By doing so, this study aims to inform the evaluation of pragmatic infection prevention policies implemented at the institutional level in vulnerable hospital populations.

**Methods and analysis:**

We will conduct a prospective, two-site interventional study in two geriatric departments of the Metz–Thionville Regional Hospital Center (Metz, France) using an alternating-period (crossover-like) design at the site level, with random allocation of the starting condition (CP first vs SP first).

Patients will be screened for ESBL-E carriage at admission and at discharge. During CP periods, contact precautions are implemented only for patients with clinically detected ESBL-E infections identified through routine clinical cultures; asymptomatic ESBL-E carriage identified through surveillance screening does not trigger contact precautions. During SP periods, patients are managed using SP alone.

The primary outcome is the incidence density of ESBL-E acquisition per 1,000 patient-days. Secondary objectives include species-specific analyses comparing ESBL-producing Escherichia coli and non-E. coli Enterobacterales. The total inclusion period is four years. Incidence rates will be compared using count regression models adapted to clustered period-based data.

## Introduction

### Background and rationale

Multidrug-resistant Gram-negative bacteria (MDR-GNB) pose a major global health challenge in the 21st century [1,2]. Since the early 2000s, the prevalence of third-generation cephalosporin–resistant Enterobacterales has steadily increased worldwide. Extended-spectrum β-lactamase–producing Enterobacterales (ESBL-E) are a major contributor to this rise [3], with numerous outbreaks reported in healthcare settings, particularly in intensive care units (ICU) and long-term care wards, leading to increased morbidity, mortality, and healthcare costs [4,5].

Initially, Klebsiella pneumoniae was the most frequently isolated ESBL-E species, but more recent epidemiological data indicate a growing burden of ESBL-producing Escherichia coli, especially in community settings [6]. In France, national surveillance data have shown a continuous increase in ESBL-E incidence, with incidence density rates now three to four times higher than those observed for methicillin-resistant Staphylococcus aureus (MRSA) [7].

Advanced age is a well-established risk factor for infection [8], largely due to immunosenescence and the high prevalence of chronic comorbidities [9–11]. A multicenter prevalence study conducted in eight French hospitals identified hospitalization in geriatric wards as a significant risk factor for ESBL-E carriage, with 28.2% of geriatric inpatients colonized [12]. Prolonged care and frequent exposure to biological fluids in geriatric settings further increase the risk of cross-transmission.

Elderly inpatients therefore present multiple risk factors for colonization with multidrug-resistant organisms (MDRO), including antibiotic exposure, colonization pressure, and organizational factors such as workload and care density. Several studies have demonstrated associations between staffing constraints, increased workload, and healthcare-associated infection risk [13,14]. Despite this, geriatric wards remain relatively understudied as potential reservoirs of ESBL-E.

Infection prevention and control (IPC) measures are central to preventing nosocomial ESBL-E transmission. Contact precautions (CP) consist of a bundle of measures aimed at limiting cross-transmission, including single-room placement or cohorting, hand hygiene, use of gowns during patient contact, and dedication of noncritical medical equipment [15]. International guidelines generally recommend CP in addition to standard precautions (SP) for patients colonized or infected with multidrug-resistant organisms [16–18].

However, growing evidence suggests that CP may not significantly reduce ESBL-E transmission compared with SP alone in certain healthcare settings [19–21]. Limited screening strategies may lead to underdetection of carriers, thereby reducing the effectiveness of targeted CP. These findings support the rationale for reinforcing universal SP for all patients, regardless of colonization status. Consistent with this approach, French national recommendations allow healthcare institutions to choose between CP- and SP-based strategies, provided that key indicators such as hand hygiene compliance and healthcare-associated infection rates are closely monitored.

The potential benefits of CP must also be balanced against unintended consequences, including increased workload and costs, reduced patient–healthcare worker interactions, and declining compliance during epidemic situations [22–26]. In resource-constrained settings, identifying effective and sustainable infection control strategies is therefore essential.

Several studies support reconsideration of CP use. Observational data from a Swiss university hospital showed low ESBL-E transmission rates in settings with high adherence to SP [27]. A cluster-randomized crossover study in Dutch hospitals demonstrated that CP in multi-bed rooms was noninferior to CP in single rooms for preventing ESBL-E transmission [28]. In addition, a non-inferiority before-and-after study conducted in an ICU at the Metz–Thionville Regional Hospital found that discontinuation of CP did not increase acquisition of MRSA or ESBL-E when high standards of hand hygiene and antimicrobial stewardship were maintained [29].

More recently, a systematic review published in 2023 concluded that discontinuation of CP for patients colonized or infected with ESBL-E had minimal clinical impact and could be safely implemented in acute, noncritical adult care wards. Importantly, pediatric and geriatric settings were not included in these analyses, and no validated guidelines currently explicitly permit CP discontinuation for geriatric inpatients with ESBL-E [30].

To the best of our knowledge, no previous study has evaluated the non-inferiority of standard precautions compared with contact precautions in geriatric care. The GERSP study is therefore the first to address this critical evidence gap in this specific clinical context.

### Study objectives and hypotheses

The primary objective of this study is to assess whether SP alone are non-inferior to clinically triggered CP with respect to limiting cross-transmission of ESBL-producing Enterobacterales in acute geriatric care units.

We hypothesize that discontinuation of CP will not result in a clinically relevant increase in hospital-acquired ESBL-E. Given that the main reservoir of ESBL-E is the gastrointestinal tract, key preventive measures focus on safe handling of excreta and appropriate hand hygiene, which are core components of SP.

The secondary objective is to assess whether transmissibility differs by ESBL-E species, with the hypothesis that cross-transmission is lower for ESBL-producing Escherichia coli than for non-E. coli Enterobacterales.

## Methods and analysis

### Study Setting

The study is conducted at the Metz–Thionville Regional Hospital Center, a bi-site tertiary care institution located in Metz and Thionville (approximately 30 km apart), serving a catchment population of about 600,000 inhabitants.

Two acute-care geriatric departments participate in the study. The geriatric department of Mercy Hospital comprises four units of 11 beds each, and the geriatric department of Bel-Air Hospital comprises two units with 15 and 23 beds, respectively. Both departments provide comparable acute geriatric care and admit older adults from the same hospital catchment area.

### Study design

This is a prospective, bi-site interventional study using an alternating-period (crossover-like) design at the site level.

Each participating site alternates between two predefined infection prevention and control (IPC) strategies—contact precautions (CP) and standard precautions (SP)—across successive study periods. The initial IPC strategy (CP first or SP first) is randomly assigned at the site level.

**Fig 1:**
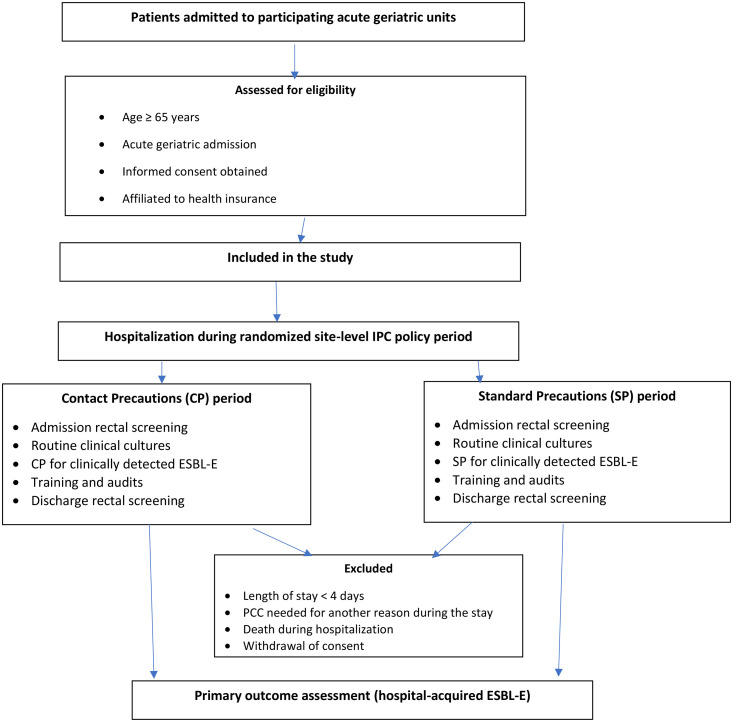
Planned CONSORT participant flow diagram for the GERSP study.

This pragmatic design was chosen to evaluate IPC policies under real-world conditions while accounting for potential seasonality and operational constraints. The overall study design, including the planned participant flow, is illustrated in Fig 1.

### Patient and public involvement

No patients or members of the public were involved in the design, conduct, reporting, or dissemination plans of this research protocol. The study was designed by clinicians and researchers based on current evidence and institutional priorities in IPC.

### Participants

#### Inclusion criteria.

Patients are eligible for inclusion if they meet all of the following criteria:

- age ≥ 65 years;
- hospitalization in a participating geriatric unit during a study period;
- affiliation to a social security system;
- written informed consent obtained within 48 hours of admission, either from the patient or a legal representative.

#### Exclusion criteria.

Patients are excluded if they meet any of the following criteria:

- conditions requiring mandatory contact precautions regardless of study period, including carbapenemase-producing Enterobacterales, glycopeptide-resistant *Enterococcus*, *Clostridioides difficile* infection, scabies, or other multidrug-resistant organisms requiring CP according to national guidelines;
- hospital stay shorter than four days;
- withdrawal of consent;
- death during hospitalization.

### Intervention and infection prevention policies

**Fig 2:**
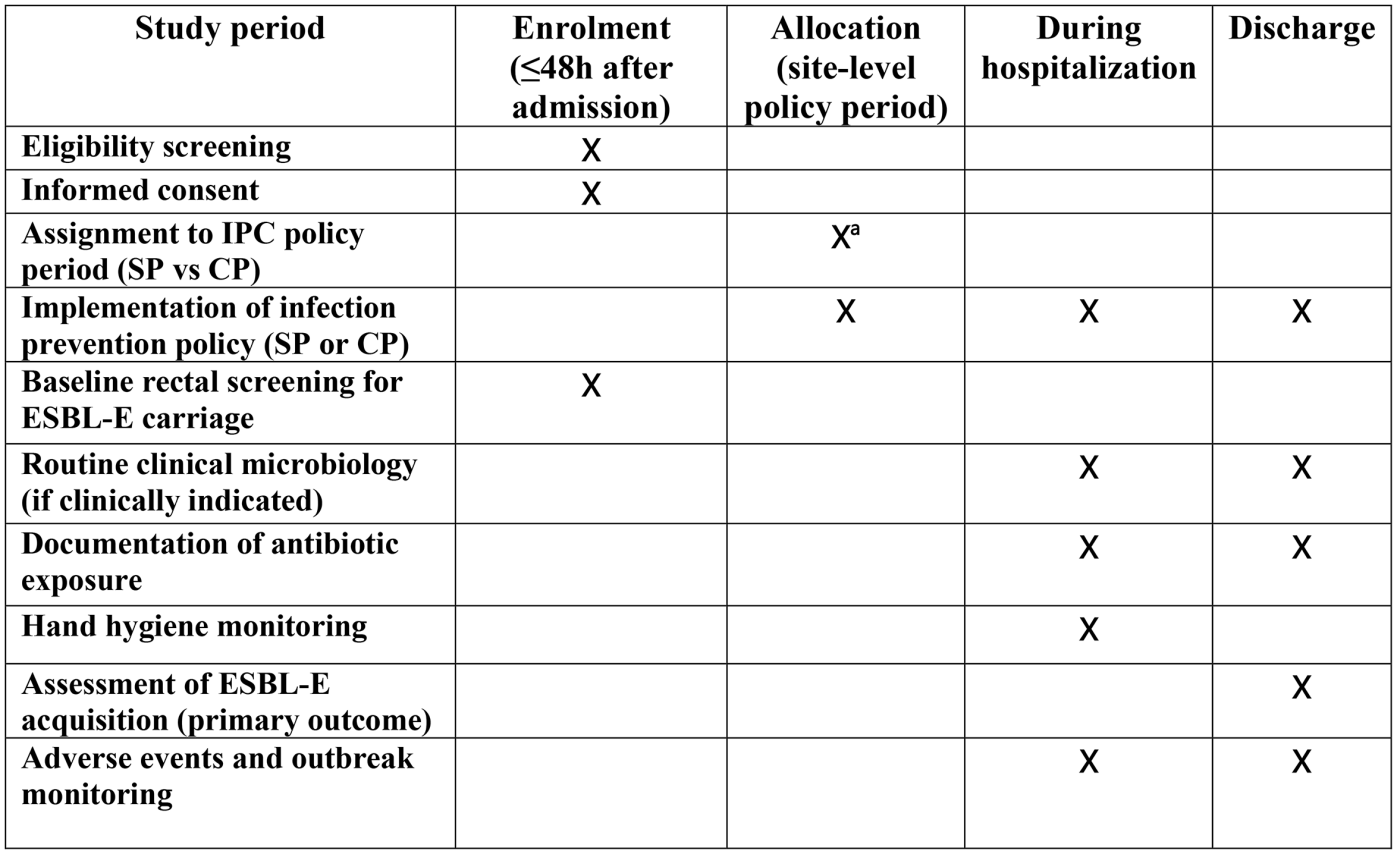
Schedule of enrolment, interventions, and assessments (GERSP study).

^a^ Allocation occurs at the site level according to the randomized order of study periods (SP first vs CP first). Participants are exposed to the infection prevention policy in effect during their hospitalization.

The detailed schedule of enrolment, interventions, and outcome assessments in accordance with SPIRIT recommendations is presented in Fig 2.

Two IPC strategies are compared (Table 1):

**Table 1:** Description of infection prevention policies during CP and SP periods.

	“CP” period	“SP” period
ESBL-E surveillance screening	Rectal screening at admission and discharge (results not disclosed to ward staff)	Rectal screening at admission and discharge (results not disclosed to ward staff)
Clinical microbiological sampling	Performed when infection is suspected, according to routine practice	Performed when infection is suspected, according to routine practice
Management of clinically detected ESBL-E infection	Contact precautions implemented in addition to standard precautions	Standard precautions only
Training and support	Training and support on SP and CP provided by the infection control team	Training and support on SP and CP provided by the infection control team
Process evaluation	Audits and interviews to assess hand hygiene practices	Audits and interviews to assess hand hygiene practices

#### CP periods.

During CP periods, patients are managed according to SP. In addition, CP (use of gowns, dedicated equipment, and patient cohorting or single-room placement when indicated) are implemented **exclusively** for patients with clinically diagnosed ESBL-E infections identified through routine clinical microbiological cultures (e.g., urine or blood cultures).

Surveillance rectal screening for ESBL-E carriage is performed at admission and discharge for research purposes only. Results of these screenings are not disclosed to ward staff, are not recorded in the electronic medical record, and do not trigger implementation of CP.

#### SP periods.

During SP periods, all patients are managed using SP alone, including hand hygiene, use of personal protective equipment according to anticipated exposure, and environmental cleaning.

### Co-interventions

Throughout the study, indications for CP unrelated to ESBL-E (e.g., carbapenemase-producing Enterobacterales, glycopeptide-resistant *Enterococcus*, *Clostridioides difficile* infection, scabies) are applied identically during both CP and SP periods, in accordance with institutional and national guidelines. Airborne and droplet precautions are implemented whenever clinically indicated, regardless of study period.

To ensure quality support from the IPC team, the two sites will be opened consecutively rather than simultaneously. The periods will be defined as follows, in order to account for potential seasonality (Fig 2):

at **Bel Air:** March–September, 2023 (**1st study period**), April–October, 2024 (**2nd study period**), April–October, 2025(**3rd study period**), April–October, 2026 (**4th study period**)
at **Mercy:** October 2023 – March, 2024 (**1st study period**), October 2024 – March, 2025 (**2nd study period**), October 2025 – March, 2026 (**3rd study period**), October 2026 – March, 2027 (**4th study period**)

One site will begin with the CP period and the other with the SP period. Throughout the study, indications for CP unrelated to ESBL-E (e.g., carbapenemase-producing Enterobacterales, glycopeptide-resistant Enterococcus, Clostridioides difficile infection, scabies) are applied identically during both periods, in accordance with institutional and national guidelines. Similarly, airborne and droplet precautions are implemented whenever clinically indicated, regardless of study period.

### Outcomes

#### Primary outcome.

The primary outcome is the incidence density of hospital-acquired ESBL-E per 1,000 patient-days, defined as ESBL-E detection at discharge following a negative admission screening.

#### Secondary outcomes.

Secondary outcomes include:

- species-specific incidence density of hospital-acquired ESBL-producing *Escherichia coli*;
- species-specific incidence density of hospital-acquired ESBL-producing Enterobacterales other than *E. coli*.

#### Exploratory outcomes.

Exploratory outcomes include hand hygiene compliance rates, compliance with personal protective equipment donning and doffing procedures, and patient-reported anxiety and depression assessed using the Hospital Anxiety and Depression (HAD) scale or a Likert scale when applicable.

### Randomization

The order of implementation of the two study periods (CP and SP) is randomly assigned at the site level. Each participating site is allocated to one of two possible sequences (CP followed by SP, or SP followed by CP) using simple randomization with a block size of two, to ensure different initial sequences across sites.

The randomization sequence is generated by the methodologist of the Clinical Research Support Platform at the Metz–Thionville Regional Hospital Center and stored in a secure electronic file. The allocation sequence is communicated to the investigators prior to study initiation.

**Fig 3:**
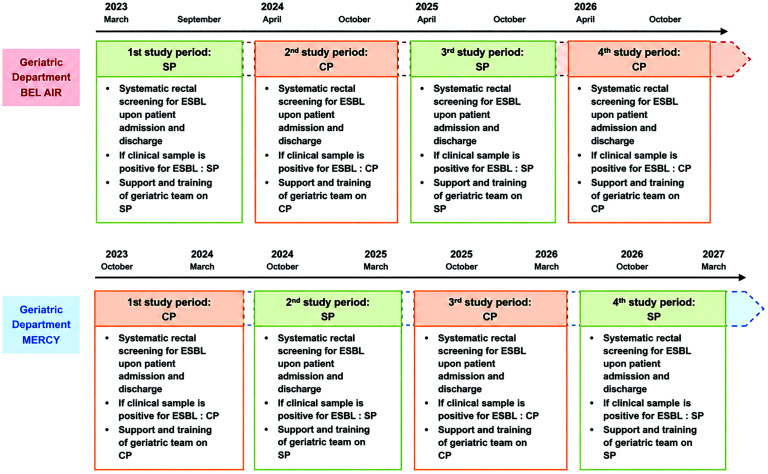
Flowchart of study procedures.

The organization of the intervention according to study site and randomized period order is illustrated in Figure 3.

#### Blinding organization of surveillance screening results.

Rectal screening for ESBL-E carriage performed at admission and discharge is conducted exclusively for research purposes. Results of these surveillance screenings are not communicated to ward staff during either study period. This approach is intended to prevent behavioral changes that could bias comparisons between infection prevention policies.

Specifically, during CP periods, disclosure of surveillance results could lead to implementation of CP beyond routine practice, whereas during SP periods, knowledge of colonization status could increase vigilance and artificially reduce cross-transmission.

Surveillance screening results are therefore accessible only to the microbiology laboratory, the clinical research support platform, and the IPC team. Results of rectal swabs are not recorded in the electronic medical record and are documented solely in the study case report form (CRF).

Importantly, results of routine clinical microbiological cultures (e.g., urine or blood cultures performed for suspected infection) are communicated to clinical teams as usual and guide antimicrobial prescribing and patient management. Only surveillance rectal screening results performed for research purposes are concealed from ward staff.

Ward staff and patients cannot be blinded to the IPC policy implemented during each period. However, patients are not informed of surveillance screening results, as these are not used for clinical decision-making.

Unblinding of surveillance results occurs only in the event of detection of emerging extensively drug-resistant bacteria (eXDR), in accordance with national “search and isolate” recommendations. In such cases, CP are immediately implemented as required for patient safety.

### Sample size

Based on a study conducted in an acute geriatric unit at a Belgian teaching hospital [31], we assumed an incidence density of 1 hospital-acquired ESBL-E acquisition per 1,000 patient-days during CP periods. The non-inferiority margin was set at 1 additional acquisition per 1,000 patient-days, based on a previous prospective non-inferiority study conducted in the intensive care unit of the same hospital institution evaluating discontinuation of contact precautions for MRSA and ESBL-E [29]. This threshold was considered clinically acceptable for the present pragmatic infection prevention study. Using a one-sided alpha of 5% and 80% power, a total of 6,178 patient-days were required.

After accounting for clustering using an intra-cluster correlation coefficient of 0.003 [32] and a design effect of 1.48 [33], the required sample size increased to 9,130 patient-days.

Based on an average length of stay exceeding four days, a total of 954 patients will be included to achieve the required number of patient-days, accounting for an anticipated 15% exclusion rate.

### Participant timeline

The participant timeline is detailed in Table 2.

**Table 2:** Participant timeline.

Study phase	Admission (≤48h after admission)	During hospitalisation	At discharge
Eligibility assessment	**X**		
Written informed consent	**X**		
Baseline rectal screening for ESBL-E carriage	**X**		
Routine clinical microbiology (if clinically indicated)		**X**	**X**
Documentation of antibiotic exposure		**X**	**X**
Monitoring of clinical status and adverse events		**X**	**X**
Discharge rectal screening for ESBL-E carriage			**X**
Assessment of ESBL-E acquisition (primary outcome)			**X**

### Microbiological samples

Rectal swabs for ESBL-E surveillance cultures are obtained by trained ward nurses within 48 hours of admission and at discharge. Samples are processed in local microbiology laboratories following standardized procedures, including direct plating on chromogenic agar for ESBL-E detection. Antimicrobial susceptibility testing is performed using disk diffusion and interpreted according to CASFM–EUCAST guidelines.

All ESBL-E isolates are stored and sent to the Infection Control Department of Besançon University Hospital for molecular typing using pulsed-field gel electrophoresis. Laboratory staff performing molecular analyses are blinded to study period and IPC strategy.

### Statistical methods

Patient characteristics will be summarized using means (± standard deviation) or medians (interquartile range) for continuous variables and counts (percentages) for categorical variables.

A non-inferiority analysis will be conducted to evaluate whether standard precautions are non-inferior to contact precautions with respect to the incidence density (per 1,000 patient-days) of hospital-acquired ESBL-E. The non-inferiority margin is set at Δ = 1 additional acquisition per 1,000 patient-days.

The primary analysis will compare ESBL-E acquisition incidence rates between SP and CP periods using a count regression model (Poisson or negative binomial in case of overdispersion), with log(patient-days) included as an offset. Models will include fixed effects for intervention (SP vs CP), calendar period or season, and period order. Additional adjusted analyses will include systemic antibiotic exposure during hospitalization as a covariate to account for antimicrobial pressure as a potential confounding factor. Marginal incidence rate differences (SP − CP, per 1,000 patient-days) and their one-sided 95% confidence intervals will be derived from the fitted models. Non-inferiority will be concluded if the upper bound of the one-sided 95% confidence interval is below the predefined margin.

All analyses will be performed using SAS version 9.3 (SAS Institute, Cary, NC, USA).

### Handling of missing data

Missing data will be described for each study period. Participants with missing discharge screening results will be excluded from the primary acquisition analysis. The extent of missing discharge screening data will also be reported.

### Process evaluation

Adherence to IPC practices will be assessed throughout the study. Hand hygiene compliance will be monitored by trained IPC staff using standardized direct observation methods based on the World Health Organization “Five Moments for Hand Hygiene” framework. Observations will be conducted regularly in each participating unit, across different shifts and days of the week.

Compliance with appropriate use of personal protective equipment during CP will also be assessed through direct observation and structured audits. To complement observational data and mitigate observer bias, alcohol-based hand rub consumption will be monitored at the unit level and summarized as volume per 1,000 patient-days by study period.

These process measures will be used to contextualize the interpretation of the study findings.

### Monitoring and oversight

#### Steering committee.

A Steering Committee will be established and composed of the coordinating investigator, scientific coordinators, the project manager, the study methodologist, and the occupational health physician. The Steering Committee will meet on a quarterly basis, or more frequently if required, to oversee study progress, protocol adherence, recruitment, data quality, and overall coordination of the research.

#### Monitoring committee.

An independent Monitoring Committee will be established to ensure participant safety and evaluate the need for early termination of the study. This committee will be composed of two infection prevention specialists and one geriatrician who are not affiliated with the Regional Hospital Center Metz–Thionville. The Monitoring Committee will meet quarterly and may recommend suspension or termination of the study in the event of an outbreak of clustered ESBL-E acquisition cases requiring temporary closure of admissions within a participating unit.

### Study sponsor

The Regional Hospital Center Metz–Thionville (France) is the sponsor of this study and is responsible for regulatory oversight, data management, and overall study coordination.

### Ethics statement

#### Research ethics approval.

This study received ethical approval from the Committee for the Protection of Persons Ouest II on August 31, 2022 (the French National Agency for Medicines and Health Products Safety [ANSM] was informed on the same date). It was prospectively registered on ClinicalTrials.gov (NCT05475574) and with the French national clinical trial registry (ID RCB: 2021-A02951-40).

#### Informed consent.

Written informed consent will be obtained from all eligible participants by a trained investigator or delegated study physician at each participating site. Consent will be sought within 48 hours of hospital admission, after verification of eligibility criteria and provision of detailed oral and written information about the study.

For patients unable to provide informed consent due to cognitive impairment or reduced decision-making capacity, consent will be obtained from a legally authorized representative, in accordance with French regulations. When possible, the participant’s assent will also be sought. Participants may withdraw their consent at any time without any impact on their medical care.

Participants will be allowed to withdraw from this study for any reason at any time without detriment to the provision or quality of their usual care.

#### Protocol amendments.

Any substantial protocol modification will be submitted to the sponsor and, prior to implementation, to the relevant ethics committee (CPP) and the competent authority (ANSM) in accordance with French regulations. Non-substantial amendments will be communicated to the ethics committee for information. All approved amendments will be communicated to participating investigators. If modifications affect participant care, risks, or benefits, updated information and consent forms will be provided and renewed informed consent will be obtained as appropriate.

#### Access to data.

Research participants have the right to access their personal data, to request rectification or erasure, to restrict processing, and to object to the processing of their personal data, in accordance with applicable data protection regulations. These rights may be exercised by contacting the study investigator, who will promptly inform the study sponsor.

## Discussion

### Limitations and interpretation

This pragmatic interventional study evaluates infection prevention and control policies under real-world conditions in an understudied acute geriatric setting. Several limitations should be considered when interpreting the findings.

First, the intervention evaluated in this study corresponded to an infection-triggered CP strategy rather than systematic isolation of all ESBL-E carriers. Consequently, the findings should therefore be interpreted within the context of routine clinical practice in acute geriatric care.

Second, allocation occurred at the site level, with only two participating hospital sites serving as clusters and alternating between CP and SP across study periods. This design limits statistical precision, causal inference, and the generalizability of findings to other healthcare settings. To mitigate this constraint, conservative modelling strategies were pre-specified, period and seasonality effects were accounted for, and sensitivity analyses were conducted. Estimates are therefore interpreted with appropriate caution.

Third, in the absence of systematic in-stay screening, the precise timing and potential source of ESBL-E acquisition during hospitalization could only be approximated using admission and discharge screening. Although this approach allows robust estimation of incidence density, it limits more granular analyses of transmission dynamics and short-term exposure effects.

Fourth, antimicrobial exposure and adherence to IPC practices are recognized drivers of ESBL-E acquisition. Routine clinical microbiological results appropriately guided antimicrobial prescribing, while surveillance screening results were concealed from staff in order to limit behavioural modification during the study. Patient-level antibiotic exposure during hospitalization will therefore be incorporated into adjusted analyses to account for antimicrobial pressure as a potential confounding factor. Measures of hand hygiene compliance and alcohol-based hand rub consumption were also incorporated as process indicators to contextualize the findings. However, residual confounding related to variations in antimicrobial use and prescribing practices cannot be completely excluded.

Co-interventions unrelated to ESBL-E prevention were maintained similarly across study periods in order to limit intervention bias.

Because the two study sites were implemented in a staggered rather than concurrent fashion, contemporaneous between-site temporal control is limited; analyses therefore adjust for calendar period, seasonality, and period order. Refresher training sessions were delivered at the beginning of each study period to maintain staff engagement and consistency in study procedures.

Taken together, these considerations indicate that the findings primarily inform the evaluation of discontinuing routine contact precautions for ESBL-E in favour of standard precautions alone in acute geriatric units operating under pragmatic, real-world conditions.

### Implications for practice and future research

In the context of increasing antimicrobial resistance and constrained healthcare resources, this study provides methodologically robust evidence on the comparative effectiveness of two widely used IPC policies implemented at the institutional level. If SP alone are shown to be non-inferior to clinically triggered CP for preventing ESBL-E acquisition, these findings would support a more targeted and pragmatic approach to isolation practices without compromising patient safety, particularly in geriatric care settings where prolonged hospital stays and functional dependence may amplify the unintended consequences of isolation.

From a research perspective, this study highlights the feasibility and value of pragmatic, policy-level interventional designs for evaluating IPC strategies under routine clinical conditions. Future pragmatic multicenter studies including a larger number of clusters and concurrent implementation across sites would help strengthen the generalizability and robustness of the findings.

### Dissemination

The results of the GERSP study will be disseminated through peer-reviewed scientific publications and presentations at national and international conferences in the fields of infection prevention and geriatric medicine. Study findings will also be reported in the ClinicalTrials.gov registry (NCT05475574) in accordance with registry requirements.

Upon study completion, a summary of the results will be made available to participating healthcare professionals within the involved geriatric units through internal presentations and written reports. A plain language summary of the main findings will be prepared and made accessible to participants upon request.

## Trial status

This study is currently ongoing. The first patient was included in March 2024 at Bel air hospital and the study is expected to end in March 2027 at Mercy hospital.

## Supporting information

S1 Checklist SPIRIT 2025 editable checklist. (DOCX)

S1 File 2021-A02951-40_PROTOCOLE_SS_v2.0_12.07.2024_Ger-SP_no_logo. (PDF)
