# Supplementary material for: Standard precautions versus clinically triggered contact precautions for prevention of extended-spectrum β-lactamase-producing enterobacterales in acute-care geriatric units: A prospective non-inferiority interventional study protocol (GERSP-study)
Source: PLoS One. 2026 Jul 21;21(7):e0353783. doi: 10.1371/journal.pone.0353783 (PMC13387550; doi:10.1371/journal.pone.0353783)
Supplement: S1 File — (PDF) [file pone.0353783.s002.pdf]

TITRE

IMPACT DE L'ABANDON DES PRECAUTIONS COMPLEMENTAIRES CONTACT AU PROFIT DES PRECAUTIONS « STANDARD » SUR LA TRANSMISSION DES ENTEROBACTERIES PRODUCTRICES DE Β-LACTAMASES A SPECTRE ETENDU EN GERIATRIE AIGUE

ACRONYME : Ger-SP

CODE PROMOTEUR : 2021-05-CHRMT

PROTOCOLE DE RECHERCHE IMPLIQUANT LA PERSONNE HUMAINE

NE COMPORTANT QUE DES RISQUES ET DES CONTRAINTES MINIMES

(2° de l'article L.1121-1 du Code de la sante publique)

Version n°2.0 du 12.07.2024
N° IDRCB : 2021-A02951-40
Avis favorable du CPP Ouest II le 31/08/2022
Information ANSM le 31/08/2022

PROMOTEUR

CHR METZ-THIONVILLE
🖃 : 1 allée du Château - CS 45001 - 57085 METZ Cedex 03

RESPONSABLE DE LA RECHERCHE AGISSANT POUR LE COMPTE DU PROMOTEUR/ET AUTORISE A SIGNER LE PROTOCOLE ET SES MODIFICATIONS EVENTUELLES AU NOM DU PROMOTEUR

M. Farid KOHILI
Directeurs des affaires médicales, de la recherche et de l'innovation par intérim
CHR METZ-THIONVILLE, Hôpital de Mercy
🖃 : 1 allée du Château - CS 45001 - 57 085 METZ Cedex 03
✆ : 03 87 65 54 00
✉ : projet-recherche-clinique@chr-metz-thionville.fr

COORDONNATEURS SCIENTIFIQUES

Dr. Laurie RENAUDIN
Service d'hygiène hospitalière
CHR Metz-Thionville, Hôpital de Mercy
🖃 : 1 allée du Château - CS 45001 - 57 085 Metz Cedex 03
✆ : 03 87 55 30 25
✉ : laurie.renaudin@chr-metz-thionville.fr

Dr. Mathieu LLORENS
Service d'hygiène hospitalière
CHR Metz-Thionville, Hôpital de Mercy
🖃 : 1 allée du Château - CS 45001 - 57 085 Metz Cedex 03

✉ : mathieu.llorens@chr-metz-thionville.fr

INVESTIGATEUR COORDONNATEUR

Dr. Noël BLETTNER
Service de gériatrie
CHR Metz-Thionville, Hôpital de Mercy
🖃 : 1 allée du Château - CS 45001 - 57085 Metz Cedex 03
☎ : 03 87 55 78 46
✉ : noel.blettner@chr-metz-thionville.fr

RESPONSABLE METHODOLOGIQUE

Dr. Christophe GOETZ
Plateforme d'Appui à la Recherche Clinique (PARC)
CHR Metz Thionville - Hôpital de Mercy
🖃 : 1 allée du Château - CS 45001 - 57 085 METZ Cedex 03
☎ : 03 87 55 77 37 46 / 🖷 : 03 87 55 77 64
✉ : christophe.goetz@chr-metz-thionville.fr

CHEF DE PROJET

Mme Arpiné EL NAR
Plateforme d'Appui à la Recherche Clinique (PARC)
CHR Metz Thionville - Hôpital de Mercy
🖃 : 1 allée du Château - CS 45001 - 57 085 METZ Cedex 03
☎ : 03 87 55 77 66 / 🖷 : 03 87 55 77 64
✉ : arpine.el-nar@chr-metz-thionville.fr

COMITE DE PILOTAGE

Dr. Noël BLETTNER
Dr. Laurie RENAUDIN
Dr. Mathieu LLORENS
Dr. Christophe GOETZ
Mme Arpiné EL NAR

Dr. Gaétan GENCO
Service de santé au travail
CHR Metz-Thionville, Hôpital de Mercy
🖃 : 1 allée du Château - CS 45001 - 57085 Metz Cedex 03
☎ : 03 87 55 64 30
✉ : gaetan.genco@chr-metz-thionville.fr

Liste des Investigateurs

Site 01 – CHRMT – Hopital Bel Air

Investigateur principal

Dr. Azzedine AZZEMOU
Service de gériatrie
CHR Metz-Thionville, Hôpital Bel Air
: 2 rue de Friscaty - 57126 Thionville Cedex
: 03 82 55 87 10
: azzedine.azzemou@chr-metz-thionville.fr

Investigateurs associes

Dr Abdoul Gadiri BALDE
Service de gériatrie
CHR Metz-Thionville, Hôpital de Bel Air
: 2 rue de Friscaty - 57126 Thionville Cedex
: 03 82 55 87 10
: abdoul-gadiri.balde@chr-metz-thionville.fr

Dr Hanane BEHIANI
Service de gériatrie
CHR Metz-Thionville, Hôpital de Bel Air
: 2 rue de Friscaty - 57126 Thionville Cedex
: 03 82 55 87 29
: hanane.behiani-maazouz@chr-metz-thionville.fr

Site 02 – CHRMT – Hopital de Mercy

Investigateur Principal/coordonnateur

Dr. Noël BLETTNER
Service de gériatrie
CHR Metz-Thionville, Hôpital de Mercy
: 1 allée du Château - CS 45001 - 57085 Metz Cedex 03
: 03 87 55 78 46
: noel.blettner@chr-metz-thionville.fr

Investigateurs associes

Dr. Philippe CHTOURBINE
Service de gériatrie
CHR Metz-Thionville, Hôpital de Mercy
: 1 allée du Château - CS 45001 - 57085 Metz Cedex 03
: 03 87 55 38 86
: philippe.chtourbine@chr-metz-thionville.fr

HISTORIQUE DES MISES A JOUR DU PROTOCOLE

	**DATE**	**RAISON DE LA MISE A JOUR**
1.0	30/03/2022	Demande initiale
1.1	08/06/2022	Demande d'information ou de modification pour une demande initiale d'avis auprès du CPP Ouest II<br>- Augmentation du nombre de patients inclus dans l'étude<br>- Recueil des données de comorbidités du patient via le score de Charlson
1.2	04/07/2022	Demande d'information ou de modification pour une demande initiale d'avis auprès du CPP Ouest II<br>- Suppression du questionnaire de satisfaction (QSH-45).<br>- Ajout de l'échelle d'évaluation de l'anxiété et de la dépression (échelle HAD).<br>- Randomisation des périodes d'étude.<br>- Constitution d'un comité de pilotage.
1.3	18/11/2022	Demande de Modification Substantielle N°1<br>- Déclaration de la participation d'un nouvel investigateur pour le centre 1 (Hôpital Bel Air/Thionville)<br>- Ajout de la description détaillée d'un des critères de non inclusion et d'exclusion (§ 5.2 et § 5.3)<br>- Ajout d'une information affichée destinée aux patients hospitalisés dans le service de gériatrie (§ 9.3 et annexe 10_v1.0_18.11.2022)<br>- Ajout des critères et procédures d'arrêt prématuré de la participation d'une personne à la recherche (§ 9.8)<br>- Ajout de la description détaillée du circuit de prélèvements d'échantillons biologiques dans le manuel de laboratoire (annexe 2_v1.3_18.11.2022)<br>- Correction de la grille AGGIR comprenant une liste plus détaillée que la précédente (annexe 4_v1.1_18.11.2022)<br>- Ajout du modèle d'étiquettes apposées sur les prélèvements pour typage moléculaire (annexe 8_v1.0_18.11.2022)<br>- Correction de l'échelle de satisfaction de Likert (annexe 9_v1.1_18.11.2022)

2.0	12/07/2024	Demande de Modification Substantielle n°2 :<br>- Ajout d'un investigateur associé au centre 01 Hôpital Bel Air<br>- Départ de deux investigateurs associés du centre 02 Hôpital Mercy<br>- Description détaillée des conditions de suspension de la recherche<br>- Demande de prolongation de la durée d'inclusion<br>- Changement de base de gestion des données (base MS ACCESS® à Cleanweb®)

SOMMAIRE

1 RESUME DE LA RECHERCHE 9

2 JUSTIFICATION SCIENTIFIQUE ET DESCRIPTION GENERALE 13

2.1 ETAT ACTUEL DES CONNAISSANCES 13

2.1.1 Sur la pathologie 13

2.1.2 Sur les strategies existantes pour lutter contre la transmission croisée des EBLSE 14

2.1.3 Hypothèses de la recherche et résultats attendus 18

2.1.4 Justification des choix methodologiques 18

2.2 RAPPORT BENEFICE / RISQUE 19

2.3 RETOMBEES ATTENDUES 19

3 OBJECTIFS DE LA RECHERCHE 20

3.1 OBJECTIF PRINCIPAL 20

3.2 OBJECTIFS SECONDAIRES 20

4 CONCEPTION DE LA RECHERCHE 21

4.1 TYPE D'ETUDE 21

4.2 LIEU DE LA RECHERCHE 21

4.3 SCHEMA DE L'ETUDE 21

4.4 METHODES POUR LA RANDOMISATION 21

5 CRITERES D'ÉLIGIBILITE 22

[illegible]ERES D'INCLUSION 22
5.2 CRITERES DE NON INCLUSION 22
5.3 CRITERES D'EXCLUSION 22

6 STRATEGIES COMPAREES DANS L'ETUDE 23

6.1 DESCRIPTION DES STRATEGIES COMPAREES 23
6.2 DESCRIPTION DES PRATIQUES D'HYGIENE 26
6.2.1 Les précautions « standard » (PS) 26
6.2.2 Les précautions complémentaires contact (PCC) 29
6.3 DESCRIPTION DES ACTIONS 29
6.4 INSU 30
6.4.1 Organisation de l'insu 30
6.4.2 Levée de l'insu 31

7 PROCEDURES ASSOCIEES 31

8 CRITERES DE JUGEMENT ET FACTEURS ETUDIES 31

8.1 CRITERES DE JUGEMENT 31
8.1.1 Critère de jugement principal 31
8.1.2 Critères de jugement secondaires 31
8.2 FACTEURS DE CONFUSION POTENTIELS 32

9 DEROULEMENT DE LA RECHERCHE 34

9.1 CALENDRIER DE LA RECHERCHE 34
9.2 TABLEAU RECAPITULATIF DU SUIVI PATIENT 34
9.3 ADMISSION DU PATIENT 35
9.4 DURANT LE SEJOUR 35
9.5 A LA SORTIE 36
9.6 RECUEIL DES PRELEVEMENTS 36
9.7 REGLES D'ARRET DE LA RECHERCHE 37
9.8 CRITERES ET PROCEDURES D'ARRET PREMATURE DE LA PARTICIPATION D'UNE PERSONNE A LA RECHERCHE 37
9.9 CONTRAINTES LIEES A LA RECHERCHE ET INDEMNISATION EVENTUELLE DES SUJETS/PATIENTS 38
9.10 COLLECTION DES ECHANTILLONS BIOLOGIQUES 38

10 GESTION DES EVENEMENTS INDESIRABLES ET DES FAITS NOUVEAUX 39

11 ASPECTS STATISTIQUES 39

11.1 CALCUL DE LA TAILLE D'ETUDE 39
11.2 METHODES STATISTIQUES EMPLOYEES 39

12 SURVEILLANCE DE LA RECHERCHE 40

12.1 COMITE DE PILOTAGE 40
12.2 COMITE DE SURVEILLANCE 40

13 DROITS D'ACCES AUX DONNEES ET DOCUMENTS SOURCE 40

13.1 ACCES AUX DONNEES 40

IEES SOURCE 40
13.3 CONFIDENTIALITE DES DONNEES 41

14 CONTROLE ET ASSURANCE QUALITE 41

14.1 CONSIGNES POUR LE RECUEIL DES DONNEES 41
14.2 SUIVI DE LA RECHERCHE 41
14.3 CONTROLE DE QUALITE 42
14.4 GESTION DES DONNEES 42
14.5 AUDIT ET INSPECTION 43

15 CONSIDERATIONS ETHIQUES ET REGLEMENTAIRES 43

16 CONSERVATION DES DOCUMENTS ET DES DONNEES RELATIVES A LA RECHERCHE 45

17 REGLES RELATIVES A LA PUBLICATION 46

17.1 COMMUNICATIONS SCIENTIFIQUES 46
17.2 COMMUNICATION DES RESULTATS AUX PATIENTS 46
17.3 CESSION DES DONNEES 46

18 REFERENCES BIBLIOGRAPHIQUES 47

ANNEXES 1

ANNEXE 1. GRILLE D'AUDIT PULPE' FRICTION 1

ANNEXE 2. MANUEL DE LABORATOIRE 1

1. Contacts 2

2. Kits 2

3. Calendrier des prélèvements rectaux 3

4. Procédure des prélèvements rectaux 3

5. Circuit d'analyse et d'envoi des prelevements 4

ANNEXE 3. ECHELLE HAD (HOSPITAL ANXIETY AND DEPRESSION SCALE) 1

ANNEXE 4. GRILLE AGGIR 1

ANNEXE 5. SCORE DE CHARLSON : COMORBIDITÉS 1

ANNEXE 6. ORDONNANCE DE PRELEVEMENT BIOLOGIQUE SPECIFIQUE A L'ETUDE 1

ANNEXE 7. MODELE D'ETIQUETTES APPOSEES SUR ECOUVILLONS RECTAUX (ESWAB ROSE) 2

ANNEXE 8. MODELE D'ETIQUETTES APPOSEES SUR PRELEVEMENTS EBLSE POSITIFS (CRYOBILLES ET TUBES DE GELOSE) 1

ANNEXE 9. ECHELLE DE LIKERT 1

ANNEXE 10. AFFICHE PATIENT 1

ONS

ANSM	Agence nationale de sécurité du médicament et des produits de santé
AGGIR	Autonomie, gérontologie, groupes iso-ressources
BA	Hôpital Bel Air
BHRe	Bactérie hautement résistante émergente
BMR	Bactérie multi-résistante
C3G	Céphalosporine de 3e génération
CPP	Comité de protection des personnes
CNIL	Commission nationale de l'informatique et des libertés
DI	Densité d'incidence
EBLSE	Entérobactérie productrice de β-lactamase à spectre étendu
EIG	Evènement indésirable grave
EPI	Equipement de protection individuelle
FHA	Friction hydro-alcoolique
IAS	Infection associée aux soins
ICH	International conference on harmonization (Conférence internationale pour l'harmonisation)
ICSHA	Indice de consommation de solution hydro-alcoolique
IDE	Infirmier diplômé d'état
JH	Journées d'hospitalisation
HCSP	Haut conseil de santé publique
HDM	Hygiène des mains
MR	Méthodologie de référence
MY	Hôpital de Mercy
OMS	Organisation mondiale de la santé
PARC	Plateforme d'appui à la recherche clinique
PCC	Précautions complémentaires de type contact
PFGE	Pulsed field gel electrophoresis
PHA	Produit hydro-alcoolique
PROPIAS	Programme national d'action de préventions des infections liées aux soins
PS	Précautions standard
SF2H	Société française d'hygiène hospitalière
SHA	Solution hydro-alcoolique
SHH	Service d'hygiène hospitalière
TA	Température ambiante

1 RESUME DE LA RECHERCHE

**PROMOTEUR**	CHR Metz-Thionville
**COORDONNATEURS SCIENTIFIQUES**	Dr Laurie RENAUDIN<br>Service d'hygiène hospitalière<br>CHR Metz-Thionville, Hôpital de Mercy<br>🖃 : 1, allée du Château - CS 45001 - 57 085 Metz Cedex 03<br>✆ : 03 87 55 30 25<br>🖂 : laurie.renaudin@chr-metz-thionville.fr<br><br>Dr. Mathieu LLORENS<br>Service d'hygiène hospitalière<br>CHR Metz-Thionville, Hôpital de Mercy<br>🖃 : 1 allée du Château - CS 45001 - 57 085 Metz Cedex 03<br>✆ : 03 87 55 38 39<br>🖂 : mathieu.llorens@chr-metz-thionville.fr
**INVESTIGATEUR COORDONNATEUR**	Dr. Noël BLETTNER<br>Service de gériatrie<br>CHR Metz-Thionville, Hôpital de Mercy<br>🖃 : 1, allée du Château - CS 45001 - 57085 Metz Cedex 03<br>✆ : 03 87 55 78 46<br>🖂 : noel.blettner@chr-metz-thionville.fr
**TITRE**	Impact de l'abandon des précautions complémentaires contact au profit des précautions « standard » sur la transmission des entérobactéries productrices de β-lactamases à spectre étendu en gériatrie aigue.<br><br>Acronyme : Ger-SP
**JUSTIFICATION / CONTEXTE**	Les infections associées aux soins (IAS) représentent une des principales causes de morbi-mortalité dans le monde. Les entérobactéries productrices de β-lactamases à spectre étendu (EBLSE) sont devenues des micro-organismes fréquemment retrouvés dans les IAS, ce qui réduit les possibilités thérapeutiques. Pour contrôler la transmission des EBLSE au sein des hôpitaux, il est classiquement recommandé de mettre en place des précautions complémentaires de type contact (PCC) en plus des précautions « standard » (PS) pour les patients colonisés et/ou infectés.<br>Depuis plusieurs années, ces recommandations sont controversées dans la littérature. Premièrement, aucune étude de haut niveau de preuve n'a démontré l'efficacité des PCC pour limiter la transmission des EBLSE. La maitrise de la diffusion des EBLSE reposant sur un ensemble de stratégies de prévention (« bundle »), l'impact individuel des PCC peut difficilement être estimé. En situation endémique, les PCC peuvent être difficiles à mettre en œuvre et s'avèrent moins efficaces voire contre-productives. En effet, les soignants surinvestissent les PCC au détriment du respect des PS, socle de la prévention de la transmission croisée. Deuxièmement, des études ont décrit un risque plus élevé d'évènements indésirables chez les patients en PCC. Enfin, plusieurs études ont montré que l'abandon des PCC chez les patients porteurs d'EBLSE n'entrainait pas d'augmentation de la transmission des EBSLE. Cependant, la majorité de ces études sont observationnelles et n'étudient pas certains facteurs importants de la transmission croisée tels que la consommation d'antibiotiques, la pression

	de colonisation, l'observance des pratiques en hygiène, la charge en soins et les moyens disponibles (personnel et matériel). De plus, elles sont pour la plupart réalisées dans des services particuliers, notamment en réanimation ou n'étudient qu'une seule espèce, *Escherichia coli*.<br>A l'heure actuelle, la littérature ne permet pas de trancher sur la stratégie à adopter pour limiter la diffusion des EBSLE.
**OBJECTIFS**	Objectif principal<br>Démontrer que les précautions « standard » (PS) seules ne sont pas inférieures aux précautions complémentaires contact (PCC) en comparant la densité d'incidence d'entérobactéries productrices de β-lactamase à spectre étendu (EBLSE) acquises en gériatrie aigue avant et après l'abandon des PCC.<br><br>Objectifs secondaires<br>1) Comparer les espèces d'EBLSE acquises en gériatrie : *Escherichia coli* versus les autres entérobactéries<br>2) Comparer les taux d'observance des pratiques en hygiène (précautions « standard » et précautions complémentaires contact) et les freins à l'hygiène des mains entre les deux périodes de l'étude<br>3) Identifier les souches par typage moléculaire<br>4) Evaluer le niveau de l'anxiété et de la dépression chez les patients, à l'aide de l'échelle HAD ou l'échelle de Likert le cas échéant (*hospital anxiety and depression scale*)
**SCHEMA DE LA RECHERCHE**	Il s'agit d'une étude prospective contrôlée randomisée (1:1) en double aveugle de type avant-après réalisée dans deux services de gériatrie (Metz et Thionville).<br><br>Pour chaque site, la période « PS » sera comparée à la période « PCC ». L'ordre des périodes (« PCC » puis « PS » OU « PS » puis « PCC ») sera attribué aux sites de façon aléatoire par tirage au sort simple, sachant qu'un site commencera par la période « PCC » et l'autre par la période « PS ».<br>Les 2 sites seront ouverts successivement et non simultanément afin de garantir un meilleur accompagnement par l'équipe d'hygiène. Pour tenir compte d'une potentielle saisonnalité, les périodes se succéderont ainsi :<br><br>07 mars – 07 octobre 2023 : 1ère période d'étude, Thionville<br>03 octobre 2023 – 03 avril 2024 : 1ère période d'étude, Metz<br>09 avril – 09 octobre 2024 : 2e période d'étude, Thionville<br>Octobre 2024 – avril 2025: 2e période d'étude, Metz<br>Avril - octobre 2025 : 3e période d'étude, Bel Air<br>Octobre 2025 – avril 2026 : 3e période d'étude Mercy<br>Avril – octobre 2026 : 4e période d'étude, Bel Air<br>Octobre 2026 – avril 2027 : 4e période d'étude, Mercy<br><br>La période « PCC » se déroulera sur 6 mois. Les mesures suivantes seront mises en place :<br>1) Prélèvements cliniques sur point d'appel clinique selon les pratiques habituelles.

	2) Mise en place des PCC en plus des PS pour tout patient porteur (infecté ou colonisé) d'EBLSE identifié à la suite des prélèvements cliniques<br><br>3) Réalisation systématique de dépistages rectaux à l'entrée et à la sortie des patients à la recherche d'EBLSE (résultats non communiqués aux cliniciens, aux soignants, ni aux patients – double aveugle). Les résultats de ces dépistages ne conduiront donc pas à la mise en place de PCC. Il n'y aura pas de changement de pratiques par rapport aux pratiques actuelles.<br><br>4) Accompagnement, sensibilisation et formation des équipes sur les PS et les PCC organisées par le service d'hygiène<br><br>5) Evaluation de l'observance, de l'observance déclarée et des freins à la friction hydro-alcoolique des mains au moyen d'audits et d'entretiens individuels<br><br>La période « PS » se déroulera sur 6 mois. Les mesures suivantes seront mises en place :<br>1) Abandon des PCC au profit des PS seules pour les patients porteurs (infectés ou colonisés) d'EBLSE<br>2) Les autres mesures mises en place durant la période « PCC » restent inchangées. Le maintien de l'aveugle permet de ne pas lever le niveau de vigilance lors de la prise en charge d'un patient connu porteur d'EBLSE
**CRITERES D'INCLUSION**	- Patient majeur > 65 ans<br>- Patient hospitalisé en gériatrie aigue pendant la période de l'étude<br>- Consentement libre et éclairé obtenu auprès du patient (ou de la personne de confiance, tuteur ou curateur le cas échéant) dans les 48h suivant son admission en hospitalisation dans le service de gériatrie<br>- Patient affilié à un régime de sécurité sociale
**CRITERES DE NON INCLUSION**	Critères de non inclusion :<br>- Patient nécessitant des PCC pour un motif autre que EBLSE (COVID-19, PCC classique hors EBLSE, PCC *Clostridium difficile*, PCC gale et PCC BHRe)<br>- Patient sous sauvegarde de justice<br>- Personne privée de liberté<br>Critères d'exclusion :<br>- Patient nécessitant la mise en place de PCC pour un autre motif autre que EBLSE au cours du séjour (COVID-19, PCC classique hors EBLSE, PCC *Clostridium difficile*, PCC gale et PCC BHRe)<br>- Séjour inférieur à 4 jours
**TRAITEMENTS/STRATEGIES/PROCEDURES DE LA RECHERCHE**	Procédure : abandon des PCC pour tout patient infecté ou colonisé à EBLSE
**CRITERES DE JUGEMENT**	1. Critère de jugement principal :<br>Densité d'incidence des EBLSE acquises en gériatrie pour 1000 journées d'hospitalisation = nombre d'EBLSE acquises / nombre de journées d'hospitalisation * 1000

	2. Critères de jugement secondaires<br>1) Densité d'incidence des BLSE acquises par espèces (*E. coli versus* non *E. coli*) pour 1000 journées d'hospitalisation<br>2) Taux d'observance à l'hygiène des mains et taux d'observance à la mise en place et au retrait des éléments de protection individuelle (chez les patients en PS et chez les patients en PCC)<br>3) Evaluation des freins à l'observance déclarée à l'hygiène des mains par friction via l'outil Pulpe' Friction<br>4) Evaluation du niveau de l'anxiété et de la dépression chez les patients, à l'aide de l'échelle HAD (auto-questionnaire) ou de l'échelle de Likert le cas échéant.
**TAILLE D'ETUDE**	Nombre de sujets nécessaires : **954**<br>Nous faisons l'hypothèse d'une densité d'incidence de 1 EBLSE acquise pour 1000 journées d'hospitalisation dans un service de gériatrie (à partir des données de la littérature et de nos données locales). Considérant une analyse de non infériorité avec une marge de 1 pour 1000, avec un risque alpha de 5% et une puissance de 80%, il est nécessaire d'inclure 562 patients. Pour tenir compte de la corrélation intra-cluster, ce nombre est majoré à 830. Pour tenir compte des sorties d'étude (durées de moins de 4 jours ou mise en place de PCC en cours de séjour), ce nombre sera majoré de 15%. Le nombre total de patients à inclure sera ainsi de 954 (477 par période).
**NOMBRE PREVU DE CENTRES**	1 centre juridique (CHR Metz Thionville) : 2 sites d'inclusion (services de médecine gériatrique de Mercy et de Bel Air)
**DUREE DE LA RECHERCHE**	**Durée de la période d'inclusion :** 48 mois<br>**Durée de participation de chaque patient** = durée effective de séjour dans le service de gériatrie<br>**Durée totale de la recherche :** période d'inclusion + analyse des données et rédaction de l'article (6 mois) = 54 mois
**ANALYSE STATISTIQUE DES DONNEES**	Une analyse de non-infériorité sera utilisée pour évaluer la non supériorité de la densité d'incidence (pour 1000 journées d'hospitalisation) des EBLSE acquises pendant les périodes « PCC », en comparaison aux périodes « PS ». La marge de non supériorité sera fixée à 1 infection pour 1000 journées d'hospitalisation.
**RETOMBEES ATTENDUES**	**Bénéfices pour le patient :**<br>- diminution des évènements indésirables liés à la mise en place des PCC anxiété / dépression).<br>- diminution des effets indésirables liés à la moindre fréquence de visite du personnel<br><br>**Bénéfices collectifs :**<br>- diminution de la transmission manuportée des micro-organismes (SARM, BHRe, *rotavirus*, etc.) à partir de patients dont le portage n'est pas connu<br>- diminution de la charge en soins liée aux précautions complémentaires

2 JUSTIFICATION SCIENTIFIQUE ET DESCRIPTION GENERALE

2.1 *ETAT ACTUEL DES CONNAISSANCES*

2.1.1 SUR LA PATHOLOGIE

Définition des bactéries multi-résistantes (BMR)

D'après le guide « Maîtrise de la diffusion des bactéries multirésistantes aux antibiotiques » publié en 1999 par le ministère de la santé, les BMR sont des bactéries qui « du fait de l'accumulation de résistances acquises à plusieurs familles d'antibiotiques, ne sont plus sensibles qu'à un petit nombre d'antibiotiques actifs en thérapeutique» (1). Les recommandations américaines définissent la BMR comme étant « un microorganisme résistant à un ou plusieurs agents antimicrobiens » (2). Enfin, selon l'organisation mondiale de la santé (OMS), la résistance aux antimicrobiens est la « résistance d'un micro-organisme à un médicament antimicrobien auquel il était jusque-là sensible » (3).

Le concept de multirésistance aux antibiotiques est un enjeu majeur de santé publique. En effet, l'acquisition de nouvelles résistances aux antibiotiques n'est plus compensée par le développement de nouvelles molécules, laissant émerger des bactéries pan résistantes pouvant engendrer des impasses thérapeutiques.

Epidémiologie des entérobactéries productrices de β-lactamase à spectre étendu

En France, les entérobactéries productrices de β-lactamase à spectre étendu (EBLSE) sont particulièrement préoccupantes en 2021 de par leur fréquence élevée, leur potentiel pathogène, leur capacité épidémiogène et leur caractère commensal qui exposent au risque de diffusion. Ce sont des bactéries commensales, présentes au niveau du tube digestif, expliquant une durée de portage prolongé accroissant le risque de transmission croisée aux autres patients. Elles ont émergé au début des années 1980 et elles ont connu une augmentation constante depuis les années 1990 (4).

Les BLSE sont des enzymes qui inactivent la plupart des bétalactamines. Elles sont apparues dans les années 1980 (types TEM et SHV) après l'utilisation des céphalosporines de 3e génération (C3G ; notamment céfotaxime et ceftazidime). Leur déterminant génétique est plasmidique permettant leur diffusion entre souches génotypiquement différentes et entre espèces d'entérobactéries (transmission horizontale). Ces souches ont d'abord été à l'origine d'épidémies essentiellement décrites en milieu hospitalier (notamment en réanimation) impliquant le plus fréquemment les espèces *Klebsiella pneumoniae* et *Enterobacter spp* (5,6). Les mesures d'hygiène basées sur le dépistage et la mise en place de précautions complémentaires contact (PCC) permettaient alors de maitriser les épidémies. A partir des années 1990, de nouvelles enzymes ont émergé : les CTX-M. L'espèce la plus impliquée était alors *Escherichia coli* (environ 50% des EBLSE) suivi de *K. pneumoniae* (5). *E. coli* est à l'origine d'infections potentiellement graves en milieu hospitalier mais aussi de 80% des infections urinaires qui constituent l'infection communautaire bactérienne la plus fréquente. En l'espace de 5 ans, une diffusion mondiale sur un mode pandémique a été constatée. C'est devant le caractère ubiquitaire d'*E. coli* et la fréquence des infections nosocomiales et communautaires dans lesquelles cette espèce est impliquée, que la maitrise de la diffusion des BLSE est devenue un enjeu majeur de santé publique en France

En France, les données de surveillance hospitalière nationale (4) ont montré une augmentation rapide de la densité d'incidence (DI) des EBLSE issues de prélèvements à visée diagnostique, passant de 0,13 pour 1000 journées d'hospitalisation (JH) en 2002 à 0,63 pour 1000 JH en 2018. L'évolution a été plus marquée chez *E. coli* BLSE (0,02 pour

1000 JH vs 0,32 pour 1000 JH en 2002 et en 2018, respectivement) que pour les autres EBLSE d'acquisition essentiellement hospitalière comme *K. pneumoniae* et *Enterobacter. cloacae*. Cependant, depuis 2016, on a observé une stabilité voire une diminution des DI *d'E. coli* BLSE. Cette tendance est également décrite au niveau européen où la résistance aux C3G chez les souches d'*E. coli* responsables de bactériémies est passée de 11,2% en 2016 à 9,6% en 2018 (2% en 2002) (7). Malgré cette évolution favorable, la diffusion massive et rapide des EBLSE reste préoccupante et représente un enjeu majeur de santé publique, notamment parce qu'elle se produit alors que des programmes de prévention sont en place. Par ailleurs, en 2018, en France, la prévalence des entérobactéries productrices de carbapénèmases (EPC) parmi les entérobactéries reste inférieure à 1% (7). Cependant, si l'incidence des EBLSE reste élevée, un recours massif aux carbapénèmes, qui constituent le traitement de référence des EBLSE, serait susceptible de conduire à l'augmentation rapide de la prévalence des EPC parmi les entérobactéries.

2.1.2 SUR LES STRATEGIES EXISTANTES POUR LUTTER CONTRE LA TRANSMISSION CROISEE DES EBLSE

La dissémination des EBLSE est la conséquence de deux déterminants : la pression de sélection exercée par les antibiotiques qui est responsable de l'émergence des EBLSE et la transmission croisée qui est responsable de la diffusion des EBLSE.

La lutte contre les EBLSE s'articule donc autour de deux axes : le bon usage des antibiotiques et la maitrise des mesures barrières d'hygiène. En France, cette lutte s'intègre dans une politique globale de prévention des infections liées aux soins (IAS) et de maîtrise de la résistance aux antibiotiques encadrée principalement par le programme national d'actions de prévention des infections associés aux soins (PROPIAS) publié en juin 2015 par le ministère des affaires sociales, de la santé et des droits des femmes (8).

Le bon usage des antibiotiques

La pression de sélection antibiotique est définie selon Santé Publique France par « la présence répétée d'antibiotique perturbant le métabolisme d'une souche bactérienne et favorisant les mutations et les échanges plasmidiques responsables d'acquisition de résistances aux antibiotiques ». Afin de réduire cette pression de sélection, il est nécessaire de progresser dans le « bon usage des antibiotiques » privilégiant l'utilisation de molécules exerçant le plus faible pouvoir sélectionnant sur les BMR et uniquement lorsque cela est nécessaire, mais aussi de limiter la surconsommation décrite en France depuis le début des années 2000 (9).

Les mesures d'hygiène

La transmission croisée est définie par la diffusion d'un micro-organisme d'une personne à une autre par l'intermédiaire d'un vecteur, essentiellement les mains et l'environnement. Ce phénomène repose sur une chaine épidémiologique qui comprend un réservoir (humain, environnement, animal), un mode de transmission (ici essentiellement par contact), une porte d'entrée (effraction cutanée, muqueuses, dispositifs invasifs) et un hôte réceptif (facteurs de risque). Sa maitrise repose sur des mesures d'hygiène définies par les différentes recommandations du haut conseil de santé publique (HCSP) et de la société française de l'hygiène hospitalière (SF2H) (10–13). Elles s'échelonnent sur 3 niveaux : les

précautions standard, les précautions complémentaires d'hygiène et les précautions spécifiques appliquées pour les bactéries hautement résistantes émergentes (BHRe – non concernées par cette étude).

Les précautions standard (PS) représentent les premières mesures « barrières » à mettre en œuvre. Elles sont le socle indispensable de la prévention de la transmission croisée et du risque infectieux. Elles doivent être appliquées par tout soignant pour tout patient quel que soit son statut infectieux (connu ou présumé) et le lieu de sa prise en charge (11). Elles s'articulent autour de six axes.

L'hygiène des mains (HDM) est le 1er axe des PS. Le rôle des mains comme vecteur de micro-organismes a largement été démontré ainsi que l'efficacité d'une bonne HDM sur la réduction de la transmission croisée ou les taux d'infections nosocomiales (14). La technique d'HDM à privilégier aujourd'hui est la friction hydro-alcoolique (FHA). Il est démontré que cette technique est bien tolérée par les équipes soignantes et augmente l'observance à l'HDM par rapport au lavage simple (15,16). Les indications à l'HDM sont décrites dans le document de l'OMS « Les 5 indications à l'hygiène des mains » : avant et après tout contact avec le patient, après tout contact avec son environnement proche ; avant tout geste aseptique, après un geste avec un risque de contact avec du liquide biologique.

Le 2ème axe est la protection de la tenue et du visage par le port d'équipements de protection individuelle (EPI). Cette mesure est indiquée en cas de risque de projection, d'aérosolisation ou de contact avec des liquides biologiques, la peau lésée ou les muqueuses. Les EPI, et particulièrement les gants, doivent être changés entre deux patients et deux activités.

Le 3ème axe est l'hygiène respiratoire. Cette mesure permet de limiter la transmission de micro-organisme transmis par voie respiratoire. Elle comprend le port de masque chirurgical pour toute personne (professionnels, patients, visiteurs, prestataires, etc.) présentant des signes cliniques de type respiratoire ; l'utilisation de mouchoirs à usage unique ; la limitation des projections naso-pharyngées en éternuant ou toussant dans son coude et l'hygiène des mains en cas de contact avec des sécrétions respiratoires. Actuellement, le port d'un masque chirurgical a été rendu obligatoire y compris en absence de signes cliniques en lien avec les gestes barrières à appliquer dans le cadre de la crise pandémique du COVID-19.

Le 4ème axe est la gestion des excréta. Il s'agit de la manipulation et de l'élimination des excréta (selles, urines, vomissures), principalement chez les patients non automnes. Il est recommandé d'utiliser les EPI nécessaires et d'effectuer une HDM lors de ces soins. La gestion des excréta est essentielle car le tube digestif est le principal réservoir des EBLSE. La stratégie de gestion des excréta doit être particulièrement définie et maitrisée par les équipes soignantes.

Le 5ème axe est la maitrise de la contamination de l'environnement (surfaces, dispositifs médicaux, linges, déchets, etc.) par un bionettoyage efficace. C'est une mesure clé pour limiter le risque de contamination des mains des soignants et la transmission croisée. Le rôle de l'environnement a été démontré lors d'épidémies notamment à certains microorganismes qui peuvent survivre plusieurs mois dans l'environnement si les conditions à leur survie sont réunies (*K. pneumoniae*, *A. baumannii*, *P. aeruginosa*, etc.) (17). Par ailleurs, Anderson *et al.* a montré dans son étude que la désinfection systématique des chambres à la sortie des patients limitait la diffusion de BMR (18).

Enfin, le 6ème axe est la prévention des accidents avec exposition au sang ou tout produit biologique d'origine humaine, en utilisant les EPI adaptés (protection du visage, de la tenue, port de gants) pour tous les soins exposant à un risque de projection ou d'aérosolisation et du matériel de sécurité en cas de manipulation d'objet perforant, coupant, tranchant.

Les précautions complémentaires de type contact (PCC) sont des mesures d'hygiène préconisées en complément des PS en cas de colonisation ou d'infection à une EBLSE. Elles sont décrites dans les recommandations d'avril 2009 de la SF2H (12). Elles ajoutent aux PS les mesures suivantes : la prise en charge du patient en chambre individuelle (ou avec un autre patient porteur de la même EBLSE), le signalement de ce portage pour tous les intervenants (notification sur la porte de la chambre et dans le dossier médical du patient), la réalisation d'une HDM avant de sortir de la chambre, le port de tablier plastique à usage unique pour tout contact direct de la tenue avec le patient, l'individualisation du matériel médical et un renforcement de la maitrise de l'environnement selon le cas (augmentation de la fréquence du bionettoyage ou changement de produit). En fonction de l'épidémiologie microbienne du secteur ou au cours d'une épidémie, l'utilité d'un dépistage systématique des EBLSE par écouvillon rectal peut être envisagée dans certains services à risque (services de soins critiques, par exemple).

Récemment, l'efficacité des PCC dans la prévention de la transmission croisée des EBLSE a été remise en cause par plusieurs études (19). De façon générale, l'efficacité des mesures d'hygiène est méthodologiquement difficile à démontrer. A ce jour, hormis pour l'HDM, l'efficacité des autres précautions n'a pas été clairement démontrée. D'un point de vue épidémiologique, l'unité d'analyse concernée n'est pas le patient en lui-même mais le service accueillant le patient, il est donc difficile de mener des études comparatives randomisées en double aveugle de bon niveau de preuve. La plupart des travaux retrouvés dans la littérature sont des études écologiques courtes (inférieures à 2 ou 3 ans) avant-après de faible niveau de preuve et ne permettant pas de prendre en compte dans l'analyse le caractère multifactoriel de la transmission des EBLSE et les principaux facteurs de confusion : la pression de colonisation, c'est-à-dire le nombre de patients porteurs de EBLSE présents dans le service, la charge en soins, la structure étudiée, l'usage des antibiotiques (20,21). La maitrise de la diffusion des EBLSE repose le plus souvent sur un ensemble de mesures (« bundle »). Avec les études disponibles à l'heure actuelle, il est difficile de connaitre précisément l'impact individuel d'une de ces mesures par rapport à une autre ou la présence d'interaction entre les différentes mesures.

Par ailleurs, des études qui questionnent l'intérêt des PCC dans certaines situations ont émergé dans la littérature.
En France, le port systématique de gants dans les PCC n'est plus recommandé depuis 2009. Il est en effet associé à une diminution de l'observance à l'HDM, notamment à l'entrée de la chambre et de nombreux mésusages (utilisation en excès, au-delà du risque d'exposition aux liquides biologiques) persistent et sont source de contamination des surfaces et de l'environnement (22).
L'ensemble du réservoir des EBLSE au sein des patients d'un hôpital ne peut pas être connu avec précision en l'absence d'une politique de dépistage systématique à l'admission des patients. Dans une enquête de prévalence dans deux hôpitaux français, la prévalence du portage d'EBLSE était de 17%; parmi ces patients seulement 34% des patients étaient connus porteurs de EBLSE, dont 20% à partir d'un prélèvement clinique et 14% par un dépistage ciblé dans les services à haut risque (23). Sauf si une politique de dépistage exhaustif est mise en place à l'admission, il n'est donc pas possible de connaitre le statut de tous les patients admis à l'hôpital. C'est pourquoi il semble plus efficace de respecter scrupuleusement les PS lors de la prise en charge de tous les patients plutôt que de placer une faible proportion de patients en PCC parce qu'ils sont connus porteurs ou infectés par une EBLSE.

Par ailleurs, les PCC viennent en complément des PS mais ne les pallient pas en cas de non-respect de ces dernières. Il semblerait que les PCC soient peu efficaces dans les services où la culture des PS est présente et où il existe un haut niveau de compliance concernant l'HDM, mais également lorsque l'observance des PS est très basse. Il semble donc nécessaire de recentrer la lutte contre la transmission des EBLSE sur la maitrise des PS afin de consolider le socle des pratiques d'hygiène plutôt que d'ajouter des mesures complémentaires sur une base fragile d'autant que l'observance des PCC diminue lorsque le nombre de patients en isolement (pression de colonisation) augmente (24).

Plusieurs études ont montré que l'abandon des PCC chez les patients porteurs d'EBLSE n'entrainait pas leur augmentation (19,24–27). Il faut cependant rester prudent face à ces études. D'une part, il s'agissait le plus souvent d'études observationnelles et mono centriques, qui pouvaient présenter des biais statistiques et méthodologiques. Par exemple, les principaux facteurs de risque de transmission croisée tels que la consommation d'antibiotiques ou le taux d'EBLSE importés (déterminant important de la pression de colonisation) ont très peu été pris en compte dans l'analyse, seules les infections à EBLSE étaient étudiées (résultats issus de prélèvements cliniques à visée diagnostique) occultant les colonisations (résultats issus de dépistage), indicateur pourtant majeur du réservoir et de la transmission croisée. D'autres part, des études récentes ont nuancé ce résultat en mettant en évidence une différence de transmissibilité entre *E. coli* BLSE et les autres entérobactéries BLSE (28,29). Dans ces études, malgré l'absence de différence significative du taux d'acquisition d'EBLSE dans le groupe PCC par rapport au groupe PS, les entérobactéries non *E. coli* acquises semblaient plus importantes lorsque les patients étaient placés dans le groupe PS. Par ailleurs, un patient porteur de *K. pneumoniae* BLSE avait 13,8% plus de risque de s'infecter avec cette bactérie qu'un patient porteur d'*E. coli* BLSE (30).

De plus, le risque d'évènements indésirables semble plus important pour les patients placés en PCC que pour les autres. Des études ont montré un impact négatif des PCC et ont décrit un risque plus élevé de dépression et d'anxiété chez le patient en PCC (31). D'autres études ont montré que le personnel prenant en charge un patient placé en PCC respectait moins les procédures de soins, entrait moins dans la chambre du patient et avait moins de contacts directs avec lui (32). Cependant, ces études sont observationnelles et comportent des facteurs confondants.

Les données de la littérature seraient en faveur d'une stratégie d'application des PS seules pour maitriser la transmission des EBLSE, notamment des *E. coli* BLSE dans certaines situations. Cependant, peu d'études de bon niveau de preuve existe, notamment face aux difficultés méthodologiques rencontrées pour contrôler tous les biais (33). En France, les recommandations ont élargi le champ des PS, les repositionnant au centre de la stratégie de lutte contre la diffusion des BMR. En 2009 (12), une stratégie exclusivement centrée sur les PS pour minimiser le risque infectieux était proposée, sous réserve du respect de certaines conditions : la mise à disposition de PHA au plus près des soins, l'observance de l'hygiène des mains élevée mesurée sur un nombre important d'observations, un niveau de consommation de PHA élevé, une proportion élevée de recours à la friction avec PHA dans les gestes d'hygiène des mains, un bon usage du port des gants et une expertise ou expérience solide de l'équipe opérationnelle d'hygiène, une connaissance solide de l'épidémiologie microbienne, basée sur des prélèvements de dépistage.

Peu d'études évaluant l'intérêt des PCC ont été menées en gériatrie. Pourtant, les personnes âgées présentent de nombreux facteurs de risque d'acquisition d'EBLSE (âge, traitement antibiotique, contact fréquent avec le milieu

hospitalier, etc.) (34,35), avec un risque élevé de développer une infection grave (36). Dans une étude de prévalence récente conduite dans 8 hôpitaux français, l'hospitalisation dans un secteur de gériatrie était un facteur de risque associé au portage d'EBLSE. Dans cette même étude, 28,2% des patients de gériatrie étaient porteurs d'EBLSE (37). Les soins réalisés au quotidien auprès des patients de gériatrie sont des soins prolongés, en contact avec des liquides biologiques dont les excréta, à haut risque de transmission croisée d'EBSLE. La manipulation des excréta, et particulièrement des selles, peut-être à l'origine de la contamination des mains et de l'environnement, favorisant la transmission croisée de micro-organismes, d'autant plus dans ces services où le réservoir semble important. Par ailleurs, en plus de la sévérité des patients, de la pression de colonisation et l'usage des antibiotiques, la transmission croisée est favorisée par des conditions locales organisationnelles telles que la charge de travail et la densité de soins. Le risque n'est pas le même dans un service de réanimation doté de moyens normés humains et matériels que dans un service de médecine classique tel que la gériatrie voire de soins de suite qui souffre actuellement en France d'absentéisme et de manque de moyens humains (38). Des travaux ont montré une corrélation entre le risque infectieux nosocomial, la charge en soins et le manque de personnel (39,40).

2.1.3 HYPOTHESES DE LA RECHERCHE ET RESULTATS ATTENDUS

Nous faisons les hypothèses suivantes :

- Les PS strictement appliquées sont aussi efficaces que les PCC pour limiter la transmission croisée d'EBLSE dans un service de gériatrie aigue. L'abandon des PCC n'engendrerait pas une augmentation d'EBLSE acquises par transmission croisée en gériatrie. Le réservoir principal des EBLSE étant le tube digestif, les mesures de prévention essentielles reposent sur la prévention du risque lors de la manipulation des excréta et l'hygiène des mains. Ces deux mesures représentent des axes majeurs des PS.
- Il existe une différence de transmissibilité entre les espèces d'entérobactéries : la transmission croisée serait moins importante chez *E. coli* BLSE que chez les entérobactéries BLSE non *E. coli.*

2.1.4 JUSTIFICATION DES CHOIX METHODOLOGIQUES

Une étude avant-après sera mise en place, comparant la période où les patients porteurs d'EBLSE seront pris en charge en PCC (période « PCC ») et la période où seules les PS seront appliqués (période « PS »). Cette étude sera réalisée sur 2 sites (Metz et Thionville) :

- Il s'agira d'une étude randomisée (1:1). Un des sites commencera par la période « PCC » et l'autre site commencera par la période « PS ». L'ordre des périodes (« PCC » puis « PS » OU « PS » puis « PCC ») sera attribué aux sites de façon aléatoire par tirage au sort simple.
- Une étude ici-ailleurs (site de Metz versus site de Thionville) n'est pas idéale dans ce contexte car le profil des patients et les habitudes d'équipes pourraient être différents d'un service à l'autre et engendrer un biais significatif. Une étude multicentrique impliquant de nombreux centres serait là encore nécessaire.
- Nous organiserons une ouverture successive des sites et non en parallèle afin de garantir un accompagnement optimal par le service d'hygiène hospitalière (un accompagnement idéal, nécessite la présence régulière sur site d'une IDE).

- Les périodes « PCC » et « PS » seront alternées sur les deux sites (Bel Air : 1ère période d'étude : 07 mars-07 septembre 2023 ; 2e période d'étude : 09 avril 2024 – 09 octobre 2024 ; 3ème période : avril 2025 – octobre 2025 ; 4e période : avril 2026 – octobre 2026. Mercy : 1ère période d'étude :03 octobre 2023 – 03 avril 2024 ; 2e période d'étude : octobre 2024 - avril 2025 ; 3e période : octobre 2025 - avril 2026 ; 4e période : octobre 2026 – avril 2027) afin de prendre en compte la saisonnalité.

2.2 RAPPORT BENEFICE / RISQUE

Les bénéfices attendus sont les suivants :

- Bénéfice individuel : diminution des évènements indésirables liés à la mise en place des PCC (aspect psychologique).
- Bénéfices collectifs :
    - diminution de la transmission manuportée des micro-organismes à partir de patients dont le portage n'est pas connu *(*SARM, BHRe, rotavirus, etc.)
    - diminution de la charge en soins liée aux précautions complémentaires

2.3 LE RECUEIL DE SELLES PAR ECOUVILLONNAGE RECTAL REPRESENTE UN RISQUE IATROGENIQUE TRES FAIBLE. C'EST UN EXAMEN DE ROUTINE, REALISE DANS LA PRATIQUE CLASSIQUE, NE NECESSITANT PAS DE FORMATIONS COMPLEMENTAIRES. IL S'AGIT CEPENDANT D'UN EXAMEN POUVANT AVOIR UNE REPERCUSSION PSYCHOLOGIQUE SUR LE PATIENT (INVASIF, RECTAL). RETOMBEES ATTENDUES

La maitrise des PS permettra la diminution des infections associées aux soins liés aux micro-organismes multi-résistants mais également à ceux responsables d'épidémies en gériatrie.

L'abandon des PCC peut présenter plusieurs avantages :

- On observe un surinvestissement des PCC en dépit du respect des PS par les professionnels de santé, par méconnaissance ou mauvaise perception du risque. Ce comportement engendre la mise en place de mesures inadaptées limitant la maitrise du risque infectieux, et le plus souvent chronophage (la mise en place de tous les EPI disponibles avant d'entrer dans la chambre d'un patient par exemple). L'abandon des PCC repositionnerait les PS au centre de la lutte contre la transmission croisée et serait un gain de temps dans la pratique.
- L'économie engendrée par l'abandon des PCC a déjà été abordée dans quelques études (41) (notamment le gain lié aux EPI non utilisés) mais aucune étude médico-économique prenant en compte tous les aspects n'a été à notre connaissance menée à ce jour. L'économie financière des EPI non utilisés pourrait paraitre dérisoire mais au lendemain de la crise sanitaire du COVID-19 responsable de tensions d'approvisionnements de matériel, la disponibilité des EPI non utilisés pour les PCC n'est pas à négliger.
- La diminution des évènements indésirables, notamment psychologiques, chez les patients en PCC.

3 OBJECTIFS DE LA RECHERCHE

3.1 OBJECTIF PRINCIPAL

Démontrer que les précautions « standard » seules (PS) ne sont pas inférieures aux précautions complémentaires contact (PCC) en comparant la densité d'incidence d'entérobactéries productrices de β-lactamase à spectre étendu (EBLSE) acquises en gériatrie aigue avant et après l'abandon des PCC.

3.2 OBJECTIFS SECONDAIRES

Les objectifs secondaires de l'étude sont :

- Comparer les espèces d'EBLSE acquises en gériatrie aigue : *Escherichia coli* versus les autres entérobactéries.
- Comparer les taux d'observance des pratiques en hygiène (PS et PCC) et les freins à l'hygiène des mains entre les deux périodes de l'étude
- Identifier les souches par typage moléculaire
- Evaluer le niveau de l'anxiété et de la dépression chez les patients, à l'aide de l'échelle HAD (annexe 3 : auto-questionnaire)

4 CONCEPTION DE LA RECHERCHE

4.1 TYPE D'ETUDE

Il s'agira d'une étude épidémiologique prospective bi-centrique comparative de type avant-après mise en place dans le service de gériatrie aigue de Mercy (site de Metz) et dans le service de gériatrie aigue de Bel Air (site de Thionville), au CHR Metz-Thionville.

4.2 LIEU DE LA RECHERCHE

Le service de gériatrie aigue de Mercy est un service de 4 secteurs de 11 lits chacun. En 2020, le nombre d'entrée était de 1675, et le nombre de journées d'hospitalisation de 15264. La durée moyenne de séjour était de 9,1 jours en 2020.
Le service de gériatrie aigue de Bel-Air est un service de 2 secteurs de 15 et 23 lits. En 2020, le nombre d'entrée était de 1308, et le nombre de journées d'hospitalisation de 13202. La durée moyenne de séjour était de 10,1 jours en 2020.

4.3 SCHEMA DE L'ETUDE

Dans cette étude, 2 périodes seront comparées :

- La période « PCC »
- La période «PS »

L'étude se déroulera au sein de deux services de gériatrie aigue du CHR Metz-Thionville : le service de Mercy et le service de Bel Air. Afin de garantir un accompagnement de qualité par l'équipe d'hygiène, les 2 sites seront ouverts successivement et non parallèlement. Les périodes seront définies comme suit, afin de tenir compte d'une potentielle saisonnalité :

- 07 mars – 07 septembre 2023 : 1ère période d'étude, Bel Air
- 03 octobre 2023 – 03 avril 2024 : 1ère période d'étude, Mercy
- 09 avril – 09 octobre 2024 : 2e période d'étude, Bel Air
- Octobre 2024 - avril 2025 : 2e période d'étude, Mercy
- Avril - octobre 2025 : 3e période d'étude, Bel Air
- Octobre 2025 – avril 2026 : 3e période d'étude Mercy
- Avril – octobre 2026 : 4e période d'étude, Bel Air
- Octobre 2026 – avril 2027 : 4e période d'étude, Mercy

Un des sites commencera par la période « PCC » et l'autre site commencera par la période « PS ». L'ordre des périodes (« PCC » puis « PS » OU « PS » puis « PCC ») sera attribué aux sites de façon aléatoire par tirage au sort simple (cf. paragraphe 4.4 ci-dessous).

4.4 METHODES POUR LA RANDOMISATION

La liste de randomisation sera établie par tirage au sort simple.

La liste de randomisation sera établie pour définir l'ordre de mise en œuvre des 2 périodes successives de l'étude (PS et PCC) pour chacun des deux sites, avec un bloc de 2 qui garantira une alternance différente entre les 2 sites. Elle sera établie par le méthodologiste de la Plateforme d'Appui à la Recherche Clinique du CHR Metz-Thionville. Elle sera conservée dans un dossier informatique protégé à la Plateforme d'Appui à la Recherche Clinique du CHR Metz-Thionville. Elle sera communiquée aux investigateurs avant le démarrage de l'étude.

5 CRITERES D'ÉLIGIBILITE

5.1 *CRITERES D'INCLUSION*

- Patient majeur > 65 ans
- Patient hospitalisé en gériatrie aigue durant la période de l'étude
- Consentement libre et éclairé obtenu auprès du patient (ou de la personne de confiance, tuteur ou curateur le cas échéant) dans les 48h suivant son admission en hospitalisation dans le service de gériatrie
- Patient affilié à un régime de sécurité sociale

5.2 *CRITERES DE NON INCLUSION*

- Patient nécessitant des PCC pour un motif autre que EBLSE :
  - COVID-19
  - PC contact classique hors EBLSE
  - PC contact *Clostridium difficile*
  - PC contact gale
  - PC contact BHRe
- Patient sous sauvegarde de justice
- Personne privée de liberté

5.3 *CRITERES D'EXCLUSION*

- Patient nécessitant la mise en place de PCC pour un motif autre que EBLSE au cours du séjour :
  - COVID-19
  - PC contact classique hors EBLSE
  - PC contact *Clostridium difficile*
  - PC contact gale
  - PC contact BHRe
- Séjour inférieur à 4 jours, car le risque d'acquisition d'EBLSE en moins de 4 jours est négligeable (13)

6 STRATEGIES COMPAREES DANS L'ETUDE

6.1 DESCRIPTION DES STRATEGIES COMPAREES

Période « PCC »	Période « PS»
**Recherche de la présence d'EBLSE selon les pratiques habituelles :**<br>**- dans le cadre d'une suspicion d'infection → prélèvement clinique**<br><br>**→ Mise en place des PCC en plus des PS pour tout patient présentant un prélèvement positif à EBLSE**	Recherche de la présence d'EBLSE selon les pratiques habituelles :<br>- dans le cadre d'une suspicion d'infection → prélèvement clinique<br><br>→ **PS seules** pour tout patient présentant un prélèvement positif à EBLSE **(= abandon des PCC)**
**Réalisation systématique de dépistages rectaux à l'admission et à la sortie des patients à la recherche d'EBLSE**<br><br>**→ Résultats non communiqués aux cliniciens et aux soignants pour ne pas engendrer une modification des pratiques.**	Réalisation systématique de dépistages rectaux **à l'admission et à la sortie** des patients à la recherche d'EBLSE<br><br>**→ Résultats non communiqués aux cliniciens et aux soignants pour ne pas élever la vigilance.**
**Accompagnement, sensibilisation et formation des équipes sur les PS et les PCC organisés par le service d'hygiène**	Accompagnement, sensibilisation et formation des équipes sur les PS organisés par le service d'hygiène
**Evaluation des pratiques au moyen d'audits et entretiens individuels pour identifier les freins à l'hygiène des mains**	Evaluation des pratiques au moyen d'audits et freins à l'hygiène des mains

Durant les 2 périodes de l'étude, les autres indications à la mise en place des PCC (présence de BHRe, infection digestive à *Clostridium difficile*, etc.) sont maintenues (12,13). De même, les autres précautions complémentaires (air et gouttelettes) sont mises en œuvre lorsque le statut du patient le nécessite (42).

L'organisation des mesures en fonction des sites de l'étude et de l'ordre des périodes déterminés par tirage au sort est décrite dans le schéma suivant :

1ère possibilité (en fonction du tirage au sort) :

Bel Air (Thionville) → 1ère période = « PCC », 2e période = « PS », 3e période = « PCC » et 4e période = « PS »

Mercy (Metz) → 1ère période = « PS », 2e période = « PCC », 3e période = « PS » et 4e période = « PCC »

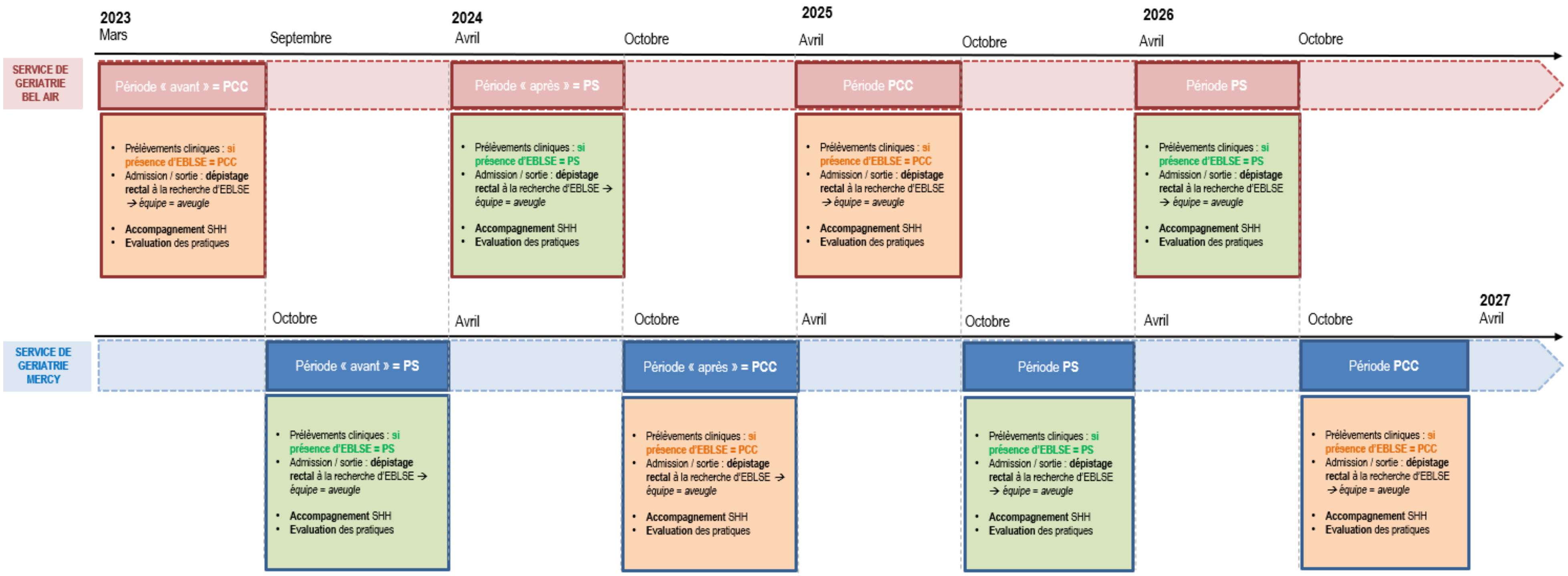

Figure 1 : organisation des mesures en fonction des sites de l'étude et de l'ordre des périodes – 1ère possibilité

2e possibilité (en fonction du tirage au sort) :

Bel Air (Thionville) → 1ère période = « PS », 2e période = « PCC », 3e période = « PS » et 4e période = « PCC »

Mercy (Metz) → 1ère période = « PCC », 2e période = « PS », 3e période = « PCC » et 4e période = « PS »

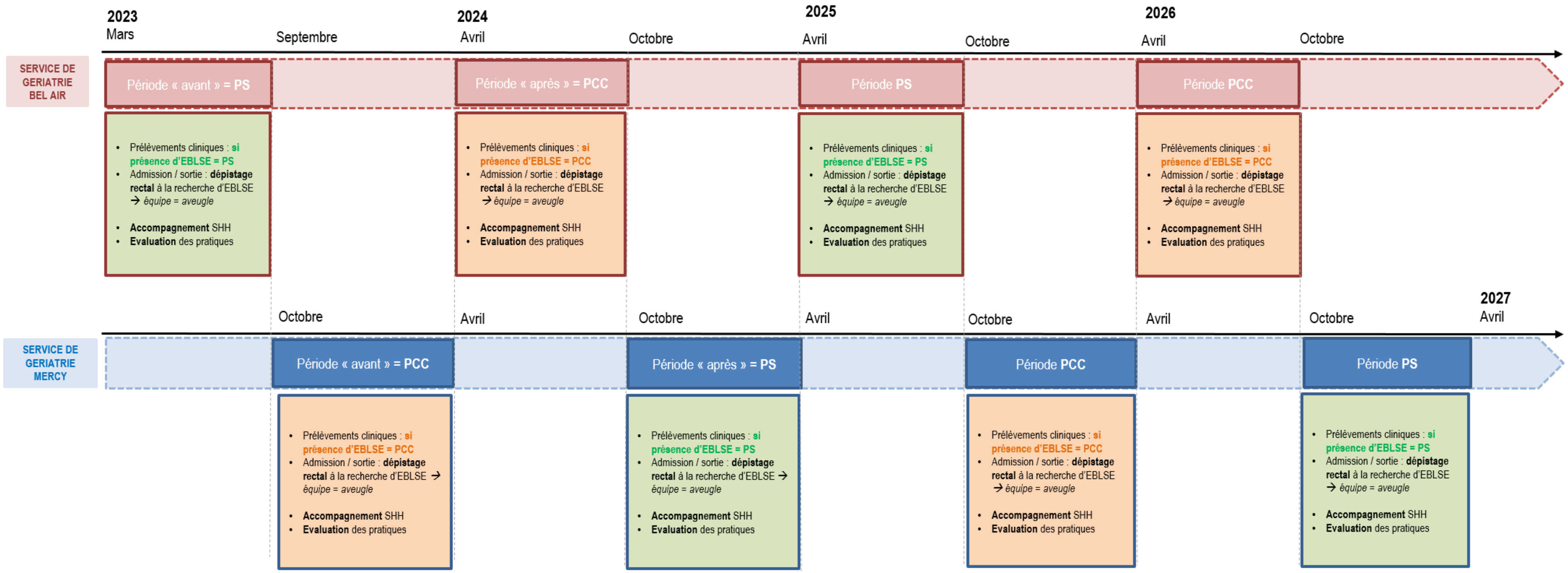

Figure 2 : organisation des mesures en fonction des sites de l'étude et de l'ordre des périodes – 2e possibilité

6.2 DESCRIPTION DES PRATIQUES D'HYGIENE

6.2.1 LES PRECAUTIONS « STANDARD » (PS)

Les PS sont décrites dans les recommandations de la société française d'hygiène hospitalière (SF2H), actualisées en juin 2017 (11). Elles sont un ensemble de mesures visant à réduire le risque de transmission croisée des micro-organismes entre les professionnels de santé, les patients et l'environnement. Elles sont à appliquer pour tout soin, en tout lieu, pour tout patient quel que soit son statut infectieux, connu ou présumé, et par tout professionnel de santé.
Elles se déclinent en 6 axes :

1. L'hygiène des mains (HDM)

Prérequis : lors des soins et en préalable à toute HDM : avoir les ongles courts, sans vernis, faux ongles, résine ; avoir les avant-bras dégagés ; ne pas porter de bijoux

Quand ? Une HDM est réalisée selon les 5 indications de l'OMS :

- Avant contact avec le patient,
- Avant un geste aseptique,
- Après un risque d'exposition à un produit biologique d'origine humaine,
- Après un contact avec le patient,
- Après un contact avec l'environnement du patient.

Comment ? la technique de référence est la réalisation d'une friction hydro-alcoolique (FHA) pour toutes les indications d'hygiène des mains en l'absence de souillures visibles. En cas de souillure visibles, un lavage simple des mains à l'eau et au savon est indiqué avant la réalisation d'une FHA

2. Le port des équipements de protection individuelle (EPI)

Les EPI protègent les professionnels de santé au risque d'exposition à des micro-organismes lors des contacts avec les muqueuses ou la peau lésée ou en cas de contact ou de risque de contact / de projection / d'aérosolisation de produit biologique d'origine humaine. Les EPI sont à usage unique et doivent être changés dès la fin du soin réalisé.

Prérequis : le port d'une tenue professionnelle propre, adaptée et dédiée à l'activité pratiquée

Quoi ?

- Le port de gants
    - Uniquement en cas de risque d'exposition au sang ou tout autre liquide biologique d'origine humaine, de contact avec une muqueuse ou la peau lésée ou lors des soins si les mains du soignant comportent des lésions cutanées
- La protection de la tenue

    - Utilisation d'un tablier plastique à usage unique pour tout soin mouillant ou souillant ou exposant à un risque de projection ou d'aérosolisation de produit biologique d'origine humaine
    - Utilisation d'une surblouse à manches longues à usage unique en cas d'exposition majeure aux produits biologiques d'origine humaine
- La protection du visage
    - Utilisation d'un masque chirurgical et d'une protection oculaire en cas de risque d'exposition par projection ou aérosolisation à un produit biologique d'origine humaine

3. L'hygiène respiratoire

L'objectif de ces mesures est de limiter la dissémination de micro-organismes via les sécrétions oro-pharyngées ou broncho-pulmonaires émises par voie respiratoire en cas de toux, d'expectoration, voire d'éternuement et ainsi de prévenir la transmission croisée interhumaine à partir d'une personne (professionnel et patient) qui présente une symptomatologie ORL ou respiratoire en l'absence de diagnostic établi.

<u>Quoi ?</u>

- Le port d'un masque chirurgical chez toute personne (professionnel, patient, résident, visiteur, intervenant extérieur, aidant, etc.) présentant une symptomatologie ORL et respiratoire supposée d'origine infectieuse en milieu de soin
- Utilisation d'un mouchoir à usage unique lors de toux, éternuement. Le jeter immédiatement après utilisation
- En absence de mouchoir, tousser ou éternuer dans son coude
- Réalisation d'une HDM après contact avec des sécrétions respiratoires ou des objets / surfaces contaminées
- Ne pas s'apporter les mains contaminées à la bouche, au niveau des yeux et du nez sans avoir réalisé une HDM

4. La gestion des excréta

Les excréta représentent un réservoir majeur de bactéries commensales du tube digestif, potentiellement porteuses de mécanismes de résistance aux antibiotiques. Le respect strict des mesures d'hygiène de base lors de leur gestion est indispensable pour éviter la transmission directe ou indirecte (via les mains des professionnels) des micro-organismes de patient à patient.

<u>Quoi</u> ? La gestion des excréta comprend :

- tous les soins en rapport avec leur manipulation (soins de nursing, toilette, changes, prélèvement, élimination),
- la vidange et l'entretien des contenants (urinal, bassin, seau de chaise percée, bocal, pot).

<u>Comment</u> ?

- Lors de la manipulation des excréta :
    - Le port de gants à usage unique et d'un tablier plastique à usage unique
    - L'élimination des EPI dès la fin du soin et réalisation d'une FHA après retrait des gants (lavage avec un savon doux avant la FHA, si mains souillées)

- Lors de l'élimination des excréta :
    - Les procédures manuelles de vidange et d'entretien des contenants sont évitées
    - Le rinçage des contenants est proscrit (ne pas utiliser les douches et les douchettes) en raison du risque d'aérosolisation

5. La gestion de l'environnement

Quoi ? La gestion de l'environnement comprend :

- La manipulation avec des EPI adaptés de tout matériel (dispositif médical, linge, déchets essentiellement) visiblement souillé ou potentiellement contaminé par du sang ou tout autre produit biologique d'origine humaine.
- La réalisation d'un nettoyage et/ou d'une désinfection avec une procédure appropriée des dispositifs médicaux ou matériel réutilisable
- La réalisation du nettoyage et/ou de la désinfection des surfaces, de l'environnement proche du patient et des locaux selon la fréquence et la procédure adaptées
- L'évacuation au plus près du soin dans un sac fermé selon la filière adaptée des déchets et du linge.

6. La prévention des accidents avec exposition au sang ou tout produit biologique d'origine humaine.

Il est recommandé de porter des EPI adaptés (protection du visage, de la tenue, port de gants) pour tous les soins exposant à un risque de projection ou d'aérosolisation et d'utiliser du matériel de sécurité en cas de manipulation d'objet perforant, coupant, tranchant.

Dans le contexte sanitaire de forte circulation du virus SARS-CoV2, les précautions « standard » ont été augmentées au CHR, notamment les mesures figurant dans l'axe Hygiène respiratoire, à savoir

- Port d'un masque chirurgical systématique pour tout le monde à l'hôpital, y compris en absence de signes cliniques : professionnel, patient en dehors de sa chambre, patient dans sa chambre en présence d'une autre personne, visiteur, intervenant extérieur, etc.

De plus, les mesures suivantes sont ajoutées :

- Port d'un masque FFP2 pour le professionnel en cas de réalisation d'acte à risque d'aérosolisation
- Port d'une protection oculaire lors de la prise en charge d'un patient, quel que soit son statut infectieux vis-à-vis de la COVID-19
- HDM avant de se porter les mains au visage

Ces mesures ont été mises en place pour limiter la transmission d'éventuels virus par voie respiratoire (notamment le SARS-CoV2), que le statut du patient / professionnel soit connu ou non. Nous estimons que ce contexte n'aura pas d'impact sur les résultats de notre étude car :

- Les mesures de préventions essentielles pour limiter la transmission croisée d'EBLSE (HDM et gestion des excréta) ne sont pas concernées par les mesures en place dans le cadre de la crise sanitaire.
- Les PCC ne sont pas modifiées par ce contexte.
- Nous pouvons faire l'hypothèse que les professionnels et les usagers sont sensibilisés aux mesures d'hygiène et notamment à l'HDM suite à cette crise. Le niveau d'HDM pourrait donc être important, mais constant durant

les deux périodes de l'étude puisque cette étude sera mise en place 2 ans après la première vague. De plus, des formations sont prévues lors des deux périodes pour sensibiliser les professionnels.

6.2.2 LES PRECAUTIONS COMPLEMENTAIRES CONTACT (PCC)

Les PCC sont des mesures d'hygiène préconisées en complément des PS en cas de colonisation ou d'infection à EBLSE. Elles sont décrites dans les recommandations d'avril 2009 de la SF2H (12).

Elles ajoutent aux PS les mesures suivantes :

- la prise en charge du patient en chambre individuelle (ou avec un autre patient porteur du même micro-organisme),
- le signalement de ce portage pour tous les intervenants (notification sur la porte de la chambre et dans le dossier médical du patient),
- la réalisation d'une HDM avant de sortir de la chambre,
- le port de tablier à usage unique pour tous contacts directs de la tenue avec le patient,
- l'individualisation du matériel médical, ou à défaut la désinfection rigoureuse du matériel après utilisation
- et un renforcement de la maitrise de l'environnement selon le cas.

6.3 DESCRIPTION DES ACTIONS

Formation des professionnels selon les modalités suivantes :

- Organisation d'une réunion de présentation inaugurale par service (Mercy et Bel-Air)
- Réalisation de 2 à 3 sessions de formation
  - Modalités = mises en situation
  - Objectif = 80% des professionnels formés + tous les nouveaux arrivants dans le mois qui suit l'arrivée dans le service
- Identification et formation de référents
- Public cible : personnel paramédical et médical
- Formateur : IDE du service d'hygiène hospitalière

Evaluation des pratiques selon les modalités suivantes :

- Audits de pratiques concernant l'HDM selon la méthodologie de l'OMS (43)
  - Fréquence : trimestrielle par service
  - Selon une grille institutionnelle adaptée de la méthodologie de l'OMS
  - Consiste à observer le nombre d'actions d'HDM réalisées lors de la prise en charge d'un patient
  - Calcul d'un taux d'observance : nombre d'actions d'HDM réalisées / nombre d'opportunités x 100
  - Réalisés par un/une IDE du service d'hygiène
- Audits concernant le port des EPI adapté d'une méthodologie canadienne
  - Fréquence : trimestrielle par service
  - Grille institutionnelle adaptée de la méthode canadienne décrite par Mitchell *et al*. (44)

- Consiste à observer la conformité du port et du retrait des EPI lors de la prise en charge d'un patient, y compris lors d'un soin en lien avec la gestion des excréta (change, mise en place ou retrait d'un plat bassin, utilisation d'un laveur désinfecteur de bassins)
  - Réalisés par un/une IDE du service d'hygiène
- Etude des freins à la friction hydro-alcoolique en cas de non-conformités
  - Utilisation de l'outil Pulpe' Friction du RéPIAS (outil officiel de la mission « Mains Propres »). Il s'agit d'un quick audit par observance déclarée (annexe 1) : les professionnels sont interrogés sur leurs pratiques en termes d'hygiène des mains. En cas de non-conformité, ils déclarent les raisons de la non observance.
  - Réalisée par un/une IDE du service d'hygiène

Feed-back / communication

- Les résultats des évaluations seront rendus aux équipes trimestriellement par le biais des cadres de santé

6.4 INSU

6.4.1 ORGANISATION DE L'INSU

Les résultats des dépistages rectaux réalisés spécifiquement pour la recherche à l'admission et à la sortie des patients ne seront pas communiqués aux professionnels de santé (médical et paramédical) pour les raisons suivantes :

- Lors de la période « PCC » : ne pas engendrer un changement de pratiques. En effet, ces dépistages ne sont pas recommandés en routine dans les services hors soins intensifs et réanimation (12). Le statut des patients colonisés par des BMR n'est pas connu en gériatrie aigue. Une augmentation du nombre de dépistages va engendrer une augmentation du nombre de patients positifs (colonisés) détectés et donc une augmentation du nombre de patients en PCC. Ce qui ne reflétera plus la situation réelle d'un service pratiquant les PS + PCC.
- Lors de la période « PS » : ne pas engendrer une augmentation de la vigilance. En effet, même si les patients positifs ne seront pas pris en charge en PCC, le simple fait de connaitre le statut du patient rend plus vigilant les professionnels les incitant à respecter les précautions d'hygiène. Ce changement de comportement pourrait engendrer une diminution artificielle des transmissions croisées. Ce qui ne refléterait plus la situation réelle de l'arrêt des PCC dans un service.

Seul le service d'hygiène, la plateforme d'appui à la recherche clinique (PARC) et le laboratoire de microbiologie du CHR seront destinataires des résultats.

Afin de respecter l'insu, un circuit est organisé avec le laboratoire de microbiologique du CHR et les résultats ne sont pas rendus sur le dossier patient informatisé (cf. manuel du laboratoire de l'étude, annexe 2). A savoir, l'étude propose un schéma en double aveugle, ce qui implique que les résultats de dépistage rectaux réalisés ne seront pas communiqués aux patients. Ils seront tracés dans le CRF de l'étude.

6.4.2 LEVEE DE L'INSU

L'insu est levé en cas de découverte fortuite de BHRe (= bactérie hautement résistante, différente des EBLSE, recherchée sur des dépistages rectaux), c'est-à-dire lorsqu'une BHRe est identifiée sur un dépistage rectal chez un patient non connu porteur. En effet, cette situation nécessite la mise en place de mesures spécifiques de prévention de transmission (PCC pour le patient porteur et le dépistage des patients contact) et un signalement aux tutelles (bénéfice individuel).

7 PROCEDURES ASSOCIEES

Non applicable

8 CRITERES DE JUGEMENT ET FACTEURS ETUDIES

8.1 CRITERES DE JUGEMENT

8.1.1 CRITERE DE JUGEMENT PRINCIPAL

Le critère de jugement principal sera la densité d'incidence des EBLSE acquises en gériatrie pour 1000 journées d'hospitalisation (JH) = nombre d'EBSLE / nombre de JH * 1000 (proportion exprimée en 1000 JH). La densité d'incidence sera calculée par période.

- *Numérateur = nombre d'EBLSE acquises*

- Une **acquisition d'EBLSE** est définie par un prélèvement positif chez un patient ayant un prélèvement négatif à l'admission
- Un **prélèvement positif** est défini par la présence d'au moins une EBLSE sur un prélèvement clinique (infection) ou de dépistage (colonisation)

- *Dénominateur = nombre de JH* = durées de séjour cumulées de tous les patients inclus à risque (= négatifs à l'admission). Le nombre de JH sera établi par période

<u>Les modalités de recueil des prélèvements biologiques sont décrites en détails dans le paragraphe 9.6 ci-dessous.</u>

8.1.2 CRITERES DE JUGEMENT SECONDAIRES

Les critères de jugement secondaires sont les suivants :

- La densité d'incidence des *E. coli* BLSE acquis pour 1000 JH = nombre de d'*E. coli* BLSE acquis / nombre de JH * 1000
- La densité d'incidence des autres entérobactéries BLSE acquises pour 1000 JH = nombre d'entérobactéries BLSE / nombre de JH * 1000
- Le taux d'observance des pratiques en hygiène (chez les patients en PS et chez les patients en PCC) :
    1. Taux de conformité aux audits HDM :
        - Un taux de conformité > 80% sera attendu pour considérer la pratique d'HDM maitrisée.

2. Taux de conformité aux audits mise en place et retrait des EPI
    - Un taux de conformité > 80% sera attendu pour considérer la pratique maitrisée.
3. Freins à l'hygiène des mains identifiés en cas de non observance (annexe 1 – audit Pulpe' Friction)
    - Les freins identifiés seront exprimés par pourcentage.

- Evaluation du niveau de l'anxiété et de la dépression chez les patients
    1. Utilisation de l'échelle HAD validé en Français (annexe 3), un auto-questionnaire évaluant le niveau de l'anxiété et de la dépression chez des patients hospitalisés (45), si l'état du patient le permet.
    2. Utilisation d'une échelle de Likert (annexe 9), si l'état du patient ne permet pas l'utilisation de l'échelle HAD.

8.2 FACTEURS DE CONFUSION POTENTIELS

La pression de colonisation

La notion de pression de colonisation permet de prendre en compte la présence de patients porteurs d'EBSLE dans le service. En effet, ces patients contribuent au réservoir à partir duquel le micro-organisme peut être transmis par transmission croisée à un patient non porteur. Il a été montré que plus le nombre de patients porteurs présents dans un service est important (plus la pression de colonisation est importante), plus le risque d'acquisition d'EBSLE par transmission croisée est important pour un patient admis non porteur (46–49). Torres-Gonzalez *et al*. identifie un risque de transmission croisée lorsque la pression de colonisation est supérieure ou égale à 10% (50).

Calcul de la pression de colonisation

Dans cette étude, la pression de colonisation sera calculée par période. La détermination individuelle de la pression de colonisation est complexe et ne connait pas de méthode de calcul consensuelle. Nous faisons l'hypothèse que les taux seront faible dans les deux périodes, résultat que nous avons observé lors de l'étude réalisée en réanimation (25).

<u>Calcul du numérateur</u>

Il s'agit de la somme cumulée des jours où un patient porteur a contribué à la pression de colonisation par séjour. Un patient porteur d'EBLSE contribue à la pression de colonisation tant qu'il est présent dans le service.

Exemple de contribution à la pression de colonisation en fonction de la situation :

- Un patient positif à l'admission = durée du séjour
- Un patient positif en cours de séjour = durée du séjour
- Un patient positif à la fin du séjour = durée du séjour
- Un patient négatif : nombre de jours de contribution à la pression de colonisation = 0

Cette méthode « simplifiée » entraine une surestimation du numérateur (nombre de jours où un patient porteur était présent) car un patient positif uniquement lors du dépistage de fin de séjour comptera pour la durée totale du séjour, ne pouvant pas connaitre de façon précise le moment de sa contamination. Cependant, cette surestimation n'engendrera pas de biais significatif car elle sera présente lors des 2 périodes et nous faisons l'hypothèses que le taux sera faible et comparable d'une période à l'autre.

La pression de sélection des antibiotiques

L'exposition aux antibiotiques est un facteur de risque d'émergence des bactéries multi-résistantes (6,51). L'exposition aux antibiotiques connus pourvoyeurs de résistances (notamment Augmentin, fluoroquinolones, C3G) sera étudiée en dose définie journalière (DDJ). Nous utiliserons les données de consommation d'antibiotiques du service à partir des bases de données de la pharmacie. Ces données seront établies par période.

Par ailleurs, les prescriptions antibiotiques individuelles des patients seront recueillies pour le séjour ayant fait l'objet de l'inclusion du patient et pour les hospitalisations des 6 mois précédents.

L'évaluation du degré de dépendance fonctionnelle à l'admission.

Le niveau d'autonomie est un facteur de risque d'acquisition d'EBLSE (52,53). En effet, plus un patient est dépendant, plus il nécessite des soins de nursing (gestion des excréta notamment) et plus le risque de transmission croisée d'EBLSE est important.

L'évaluation du degré de dépendance fonctionne à l'admission s'effectuera à l'aide de la grille nationale AGGIR (autonomie, gérontologie, groupes iso-ressources). La grille AGGIR se présente sous forme de questions et d'observations permettant d'évaluer les activités corporelles et mentales (dites « discriminatives ») et les activités domestiques et sociales (dites « illustratives ») (annexe 4). Les degrés de perte d'autonomie sont classés en 6 *groupes iso-ressources* (Gir). À chaque Gir correspond un niveau de besoins d'aides pour accomplir les actes essentiels de la vie quotidienne (Gir 1 : perte d'autonomie totale ; Gir 6 : parfaite autonomie).

Autres facteurs de risques

Les autres facteurs de risque d'acquisition de EBLSE étudiés seront l'âge, le sexe, la provenance du patient [EHPAD, domicile, hospitalisation (transfert), mutation interne], passage par les urgences avant admission service gériatrie, la durée de séjour, le nombre d'hospitalisations antérieures, les comorbidités étudiés par le score de Charlson (annexe 5) (57) dans les 6 mois.

9 DEROULEMENT DE LA RECHERCHE

9.1 CALENDRIER DE LA RECHERCHE

- Début des inclusions : admission des patients à partir du 07 mars 2023
- Durée de la période d'inclusion : 4 périodes x 6 mois x 2 sites successivement = 48 mois
- Durée de participation de chaque patient : durée effective de séjour dans le service de gériatrie aigue
- Durée totale de la recherche : 48 mois + 6 mois (exploitation et analyses des données, rédaction de l'article) = 54 mois

9.2 TABLEAU RECAPITULATIF DU SUIVI PATIENT

	Admission du patient	Durant le séjour	Sortie du patient
Vérification des critères d'éligibilité	✓		
Information du patient	✓		
Consentement éclairé écrit et signé (48h)	✓		
Caractéristiques de patients (âge, sexe, degré de dépendance (Grille AGGIR ; annexe 4) ; comorbidités (score de Charlson ; annexe 5)	✓		
Provenance du patient, nombre d'hospitalisations à l'entrée (dans les 6 mois)	✓		
Durée de séjour du patient			✓
Echelle HAD (annexe 3) et Echelle de Likert (annexe 9)			✓
Prélèvements microbiologiques cliniques (sang, urine, selles, expectoration, autre [1]…) **réalisés dans la pratique habituelle** + typage moléculaire (annexes 2 et 8)	**SI** suspicion d'infection [2]	**SI** suspicion d'infection [2]	
Dépistage rectal à la recherche de EBLSE [3], **réalisés dans le cadre de l'étude** (annexes 6 et 7) + typage moléculaire (annexes 2 et 8)	✓ **Systématiquement,** dans les 48h après l'admission		✓ **Systématiquement**

[1] Tout prélèvement à visée diagnostique (écouvillonnages, abcès, etc…)

[2] Résultats communiqués à l'équipe : prélèvement positif à EBLSE durant la période « PCC », → PS + PCC / prélèvement positif à EBLSE durant la période « PS » → PS.

[3] Résultats non communiqués à l'équipe (quelle que soit la période)

9.3 ADMISSION DU PATIENT

L'admission du patient est réalisée par l'équipe de médecine gériatrique. Les actions suivantes sont réalisées :

Information et recueil du consentement

Lors de l'admission, le médecin investigateur prenant en charge le patient lui remet une note d'information et répond à toutes ses questions concernant l'objectif, la nature des contraintes, les risques prévisibles et les bénéfices attendus de la recherche. Il précise également les droits du patient dans le cadre d'une recherche biomédicale et vérifie les critères d'éligibilité.

Le consentement libre et éclairé du patient (ou de son représentant légal ou de sa personne de confiance dans le cas où le patient n'est pas en capacité de donner son consentement éclairé) est recueilli par écrit par le médecin investigateur dans les 48h suivant son admission dans le service de gériatrie puis tracé dans le dossier patient informatisé. Les médecins investigateurs seront les médecins gériatres des services de médecine gériatrique.

Le patient inclus ne pourra pas participer simultanément à une autre recherche clinique.

Durant les périodes PS (abandon des PCC), une information concernant le changement de pratiques sera également publiée par le biais d'une affiche apposée dans les chambres des patients admis dans le service de gériatrie et/ou dans la salle de soins du service (cf. annexe 10 : affiche patient). Ce qui permettra d'informer l'ensemble des patients admis dans le service et pas uniquement ceux qui sont inclus dans l'étude.

Dépistage rectal

Un dépistage rectal sera effectué dans les 48h après l'admission du patient inclus dans la recherche. L'écouvillon sera envoyé au laboratoire de microbiologie du CHR. Les résultats ne seront pas communiqués aux équipes de soins, ni aux patients, seuls le service d'hygiène, la PARC et le laboratoire de microbiologie auront connaissance des résultats.

Prélèvements réalisés dans le cadre de la pratique habituelle

Des prélèvements cliniques pourront être réalisés en cas de suspicion clinique d'infection. Les résultats de ces prélèvements seront communiqués aux équipes pour prise en charge. Lors de la période « PCC », la présence d'EBLSE sur ces prélèvements engendrera la mise en place de PCC en plus des PS, lors de la période « PS » ; seuls les PS seront en place (=abandon des PCC).

9.4 DURANT LE SEJOUR

Des prélèvements cliniques pourront être réalisés en cas de suspicion clinique d'infection. Les résultats de ces prélèvements seront communiqués aux équipes pour prise en charge. Lors de la période « PCC », la présence d'EBLSE sur ces prélèvements engendrera la mise en place de PCC en plus des PS, lors de la période « PS » ; seuls les PS seront en place (=abandon des PCC), (voir paragraphe 9.6 ci-dessous).

9.5 A LA SORTIE

Un dépistage rectal sera effectué à la sortie du patient, quel que soit sa destination (retour à domicile, transfert dans un autre établissement, transfert dans un autre service du CHR). L'écouvillon sera envoyé au laboratoire de microbiologie du CHR. Les résultats ne sont pas communiqués aux équipes de soins, ni aux patients, seuls le service d'hygiène et le laboratoire de microbiologie ont connaissance des résultats (voir paragraphe 9.6 ci-dessous).

9.6 RECUEIL DES PRELEVEMENTS

Les résultats du recueil des dépistages rectaux ne seront pas communiqués ni aux cliniciens ni aux soignants, ni aux patients (étude en double aveugle). Ils ne conduiront pas à la mise en place de PCC en cas de résultat positif. Il n'y aura pas de changement de pratiques par rapport aux pratiques actuelles.

<u>Prélèvements biologiques à la recherche d'EBLSE</u> :

- Rythme des prélèvements

Dans cette étude, les prélèvements pourront être réalisés dans deux situations (cf. annexe 2) :

- Dans la pratique habituelle (= pas de changement de pratiques)
    - Prélèvement clinique devant une suspicion d'infection (urinaire, infection liée au cathéter, etc.)
    - Les résultats de ces prélèvements seront communiqués aux équipes comme habituellement
- Spécifiquement pour l'étude (prélèvement supplémentaire)
    - Dépistage par écouvillonnage rectal (colonisation digestive) à l'admission du patient dans le service et à la sortie
    - Les résultats de ces prélèvements ne seront pas communiqués aux équipes pour ne pas engendrer de changement de pratiques et de comportement

- Réalisation et technique

Un nombre total de 1908 dépistages rectaux est nécessaire pour la réalisation du protocole de recherche, à l'entrée et à la sortie d'hospitalisation des patients.

Les dépistages rectaux seront :

- réalisés par un(e) infirmier(e) diplômé(e) d'état (IDE)
- par écouvillonnage rectal (technique standardisée selon le manuel de prélèvement institutionnel) ou recueil de selles dans un pot à coproculture.
- en utilisant un écouvillon avec milieu de transport

Les prélèvements cliniques seront réalisés par les médecins ou les IDE selon la technique institutionnelle en fonction des compétences nécessaires (médicales ou paramédicales). Ils peuvent être conservés à 4°C pendant 24H avant leur acheminement au sein du laboratoire de microbiologie de Mercy pour analyse.

- Analyses des prélèvements

Les prélèvements seront analysés au laboratoire de microbiologie de Mercy selon la technique suivante :

- Isolement et détection des EBLSE sur milieux de culture chromogène sélectifs additionnés à un mélange d'antibiotiques (ChromID ESBLE – BioMérieux, Marcy-l'Etoile)
- Identification des souches par spectrométrie de masse (MALDI-TOF MS)
- Détermination de la sensibilité aux antibiotiques par diffusion en milieu solide gélosé (gélose Mueller-Hinton – MH) et lecture par automate (Sirscan, i2a Diagnostics, Montpellier). Interprétation des diamètres selon les recommandations de la CA-SFM/ EUCAST (version qui sera en vigueur lors du déroulement de l'étude).
- <u>Les échantillons prélevés seront détruits après analyse.</u>

- Typage moléculaire : Tout prélèvement positif sera envoyé au service d'hygiène hospitalière du CHU de Besançon de Pr Xavier BERTRAND pour réalisation d'un typage moléculaire.

- Les souches pures seront stockées à -80°C, les souches EBLSE positives seront ensemencées et conservées en gélose et seront envoyées semestriellement au laboratoire du CHU de Besançon par un transporteur spécialisé
- Elles seront alors analysées par électrophorèse en champ pulsé (PFGE)

9.7 REGLES D'ARRET DE LA RECHERCHE

La recherche peut être suspendue dans toutes situations ne permettant plus de garantir les bonnes pratiques cliniques et les moyens humains nécessaires pour la bonne mise en œuvre de la recherche.
Modalités de mises en œuvre :

- La demande de suspendre la recherche est proposé par le Comité de Pilotage (méthodologiste, chef de projet, investigateur coordonnateur, coordonnateur scientifique) au Comité de Surveillance qui émet un avis.
- L'avis est validé par le promoteur.

9.8 CRITERES ET PROCEDURES D'ARRET PREMATURE DE LA PARTICIPATION D'UNE PERSONNE A LA RECHERCHE

Les motifs de sortie d'étude d'un patient seront les suivants :

- Décès du patient pendant sa durée de participation
- Inclusion à tort : découverte après l'inclusion (avant ou après l'intervention) d'un critère de non inclusion ou d'exclusion
- Retrait du consentement du patient ou de son représentant légal ou de la personne de confiance désignée
- Déviation majeure au protocole

Devenir du patient : en cas d'arrêt prématuré, d'exclusion, retrait de consentement, abandon ou déviations majeures, le patient réintégrera sa filière de soins habituelle.

9.9 CONTRAINTES LIEES A LA RECHERCHE ET INDEMNISATION EVENTUELLE DES SUJETS/PATIENTS

La contrainte liée à la recherche est la réalisation de prélèvements rectaux à l'admission et à la sortie :

- Pratique non réalisée en routine dans ce service
- Acceptabilité Prélèvements entrées / sorties

Aucune indemnisation éventuelle des sujets ou des patients n'est prévue dans ce protocole de recherche.

9.10 COLLECTION DES ECHANTILLONS BIOLOGIQUES

Non applicable

10 GESTION DES EVENEMENTS INDESIRABLES ET DES FAITS NOUVEAUX

Aucune procédure de gestion des évènements indésirables graves n'est imposée par la recherche. Cependant, la déclaration des effets graves des médicaments (au centre régional de pharmacovigilance) ou des dispositifs médicaux (au correspondant local de matériovigilance) est obligatoire pour tout médecin (ou autre professionnel de santé concerné), aussi bien dans le contexte de cette recherche qu'en dehors.

11 ASPECTS STATISTIQUES

11.1 CALCUL DE LA TAILLE D'ETUDE

La littérature rapportait en 2012 une densité d'incidence attendue des EBLSE acquises dans un service de gériatrie avec PCC de 1,77 pour 1000 JH (52). En 2019, au sein des services de gériatrie du CHR, la densité d'incidence des EBLSE acquises identifiées sur des prélèvements cliniques (infection) était de 0,3 pour 1000. A partir de ces données, nous faisons l'hypothèse d'une densité d'incidence 1 EBLSE acquise pour 1000 JH pour la période avec PCC. Considérant une analyse de non infériorité avec une marge de 1 pour 1000 (marge observée pour une étude similaire en réanimation (25)), avec un risque alpha de 5% et une puissance de 80%, il est nécessaire d'inclure 6178 journées d'hospitalisation au total (3089 par période). En considérant une corrélation intra-cluster des patients de 0.003 (29) et un « design effect » de 1.48 (56), ce nombre est majoré à 9130 journées.

Lors du premier semestre 2021, la durée moyenne des séjours de plus de 4 jours dans les services concernés était de 11 jours. Ainsi il faudra inclure 830 patients pour obtenir le nombre souhaité de journées d'hospitalisation. Pour tenir compte des sorties d'étude (durées de moins de 4 jours ou mise en place de PCC en cours de séjour), ce nombre sera majoré de 15%. Le nombre total de patients à inclure sera ainsi de 954 (477 par période).

Nous nous attendons à un taux de prélèvements positifs à EBLSE de 12% à 28% selon la littérature, en population gériatrique (37,52), d'où 114 (12% de 954 patients inclus) à 267 (28% de 954 patients inclus) analyses par typage moléculaire à réaliser. Et parmi ces prélèvements, environ 3,6 % (1,77 pour 1000 JH) seraient acquises (52).

11.2 METHODES STATISTIQUES EMPLOYEES

Les données qualitatives seront décrites sous la forme d'effectifs et pourcentages, les données quantitatives sous la forme de moyenne +/- écart type, ou médiane et intervalle inter-quartiles en cas de distribution non normale. Elles seront comparées à l'aide de tests exacts de Fischer, de tests T de Student ou de tests non paramétriques de Wilcoxon respectivement.

Une analyse de non-infériorité sera utilisée pour évaluer la non supériorité de la densité d'incidence (pour 1000 JH) des EBLSE acquises pendant les périodes « PCC », en comparaison aux périodes « PS ». La marge de non supériorité sera fixée à 1 EBLSE acquise pour 1000 JH. Les densités d'incidence seront comparées à l'aide du test de Schuirmann (54).

Des biais de confusion seront recherchés concernant les variables : site d'inclusion, consommation antibiotique, pression de colonisation et taux d'observance des précautions d'hygiène. Les facteurs de confusion seront pris en compte comme suit :

- Vérifier que les facteurs de confusion sont homogènes / comparables entre les deux périodes (comparaisons bi-variées)
- En cas d'hétérogénéité et lorsque cela sera possible, des analyses en sous-groupes seront réalisées

Les analyses seront réalisées à l'aide du logiciel SAS version 9.3 (SAS Institute, Cary, NC).

Un plan d'analyse détaillé sera défini et fera l'objet d'une validation par le conseil scientifique de l'étude. Les modifications ultérieures devront intervenir avant la levée d'insu sur la base de données et seront systématiquement validées par le conseil scientifique.

12 SURVEILLANCE DE LA RECHERCHE

12.1 COMITE DE PILOTAGE

Un comité de pilotage sera constitué de l'investigateur coordonnateur, des coordinateurs scientifiques, du chef de projet, du méthodologiste ainsi que du médecin de travail. Il se réunira selon un rythme trimestriel, ou de façon plus rapprochée si nécessaire.

12.2 COMITE DE SURVEILLANCE

Un comité de surveillance composé de 2 hygiénistes et d'un gériatre hors CHR Metz Thionville sera établi afin de mettre fin à l'étude en cas de survenue d'épidémie de cas groupés d'EBSLE acquises nécessitant l'arrêt des admissions dans le service. La fréquence des réunions sera trimestrielle.

13 DROITS D'ACCES AUX DONNEES ET DOCUMENTS SOURCE

13.1 ACCES AUX DONNEES

Le promoteur est chargé d'obtenir l'accord de l'ensemble des parties impliquées dans la recherche afin de garantir l'accès direct à tous les lieux de déroulement de la recherche, aux données source, aux documents source et aux rapports dans un but de contrôle de qualité et d'audit par le promoteur.

Les investigateurs mettront à disposition les documents et données individuelles strictement nécessaires au suivi, au contrôle de qualité et à l'audit de la recherche biomédicale, à la disposition des personnes ayant un accès à ces documents conformément aux dispositions législatives et réglementaires en vigueur (articles L.1121-3 et R.5121-13 du code de la santé publique).

13.2 DONNEES SOURCE

Tout document ou objet original permettant de prouver l'existence ou l'exactitude d'une donnée ou d'un fait enregistrés au cours de la recherche est défini comme document source.

Les documents sources utilisés dans le cadre de la recherche seront :

- Le dossier médical informatisé

- Résultats des analyses microbiologiques du laboratoire de microbiologie de Mercy et du laboratoire de Besançon

13.3 CONFIDENTIALITE DES DONNEES

Conformément aux dispositions législatives en vigueur (articles L.1121-3 et R.5121-13 du code de la santé publique), les personnes ayant un accès direct aux données source prendront toutes les précautions nécessaires en vue d'assurer la confidentialité des informations relatives aux médicaments expérimentaux, aux recherches, aux personnes qui s'y prêtent et notamment en ce qui concerne leur identité ainsi qu'aux résultats obtenus. Ces personnes, au même titre que les investigateurs eux-mêmes, sont soumises au secret professionnel.

Pendant la recherche biomédicale ou à son issue, les données recueillies sur les personnes qui s'y prêtent et transmises au promoteur par les investigateurs (ou tous autres intervenants spécialisés) seront rendues anonymes. Elles ne doivent en aucun cas faire apparaître en clair les noms des personnes concernées ni leur adresse.
Les sujets seront identifiés par un numéro codé correspondant à : numéro de site – numéro d'ordre d'inclusion.

Le promoteur s'assurera que chaque personne qui se prête à la recherche a donné son accord par écrit pour l'accès aux données individuelles la concernant et strictement nécessaires au contrôle de qualité de la recherche.

14 CONTROLE ET ASSURANCE QUALITE

14.1 CONSIGNES POUR LE RECUEIL DES DONNEES

Toutes les informations requises par le protocole doivent être consignées sur les cahiers d'observation et une explication doit être apportée pour chaque donnée manquante. Les données devront être recueillies au fur et à mesure qu'elles sont obtenues, et transcrites dans ces cahiers de façon nette et lisible.
Les données erronées relevées sur les cahiers d'observation seront clairement barrées et les nouvelles données seront copiées, à côté de l'information barrée, accompagnées des initiales, de la date et éventuellement d'une justification par l'investigateur ou la personne autorisée qui aura fait la correction.

Les données de microbiologie, l'antibiothérapie et le nombre d'hospitalisations de l'étude seront directement extraites des bases de données du laboratoire, de la pharmacie et du dossier patient informatisé.

14.2 SUIVI DE LA RECHERCHE

Le suivi de la recherche sera assuré par un technicien de recherche clinique. Il sera chargé, auprès de l'investigateur coordonnateur, de :

- la logistique et la surveillance de la recherche,
- l'établissement des rapports concernant son état d'avancement,
- la vérification de la mise à jour du cahier d'observation (demande d'informations complémentaires, corrections,…),

- l'envoi des prélèvements,

Il travaillera conformément aux procédures opératoires standardisées, en collaboration avec l'attaché de recherche clinique délégué par le promoteur.

14.3 CONTROLE DE QUALITE

Un attaché de recherche clinique mandaté par le promoteur visite de façon régulière chaque site investigateur, lors de la mise en place de la recherche, une ou plusieurs fois en cours de recherche selon le rythme des inclusions et en fin de recherche. Lors de ces visites, les éléments suivants seront revus :

- consentement éclairé, (100%)
- respect du protocole de la recherche et des procédures qui y sont définies,

A ce titre, l'investigateur s'engage à mettre à la disposition de l'ARC lors de ses visites de monitoring :

- Les dossiers médicaux des patients
- Les formulaires de consentement des patients inclus

Ce monitoring permettra d'évaluer :

- La protection des personnes
- La fiabilité des données par rapport aux documents sources
- La conformité de l'essai par rapport au protocole, aux Bonnes Pratiques Cliniques et à la législation en vigueur en matière de recherches biomédicales.

Toute visite fera l'objet d'un rapport de monitorage par compte-rendu écrit.

14.4 GESTION DES DONNEES

La gestion des données est assurée par la Plateforme d'Appui à la Recherche Clinique du CHR Metz-Thionville, dans le logiciel de Data Management Cleanweb®, de la phase de collecte jusqu'à l'extraction :

- La saisie s'effectue par les formulaires du Cahier d'Observation Electronique (eCRF) implémentées dans le logiciel.
- A l'exception des données microbiologiques qui sont extraites du logiciel du laboratoire (GLIMS).
- Les données de Cleanweb® et du laboratoire sont jointes avant le gel de la base de données.
- La sauvegarde de la base de données est quotidienne, elle est assurée par le gestionnaire du logiciel Cleanweb® via un plan de continuité de service.
- Une fois le gel de base réalisé, une extraction CSV des données est fournie au statisticien.

L'exploitation statistique est réalisée par la PARC à l'aide du logiciel SAS™ (SAS Institute, USA).

14.5 AUDIT ET INSPECTION

Un audit peut être réalisé à tout moment par des personnes mandatées par le promoteur et indépendantes des responsables de la recherche. Il a pour objectif de s'assurer de la qualité de la recherche, de la validité de ses résultats et du respect de la loi et des réglementations en vigueur.

Les investigateurs acceptent de se conformer aux exigences du promoteur et à l'autorité compétente en ce qui concerne un audit ou une inspection de la recherche.

L'audit pourra s'appliquer à tous les stades de la recherche, du développement du protocole à la publication des résultats et au classement des données utilisées ou produites dans le cadre de la recherche.

15 CONSIDERATIONS ETHIQUES ET REGLEMENTAIRES

Le promoteur et l'(es) investigateur(s) s'engagent à ce que cette recherche soit réalisée en conformité avec le Code de la santé publique, ainsi qu'en accord avec les Bonnes Pratiques Cliniques (I.C.H. version 4 du 1er mai 1996 et décision du 24 novembre 2006) et la déclaration d'Helsinki (qui peut être retrouvée dans sa version intégrale sur le site http://www.wma.net).

La recherche est conduite conformément au présent protocole. Hormis dans les situations d'urgence nécessitant la mise en place d'actes thérapeutiques précis, l'(es) investigateur(s) s'engage(nt) à respecter le protocole en tous points en particulier en ce qui concerne le recueil du consentement.

Cette recherche a reçu un numéro ID-RCB sur le site de l'ANSM : https://ictaxercb.ansm.sante.fr/Public/index.php .

Cette recherche a reçu l'avis favorable du Comité de protection des personnes (CPP) Ouest II le 31/08/2022. Son résumé ainsi que l'avis du CPP ont été transmis à l'ANSM pour information le même jour.

Le CHR Metz Thionville, promoteur de cette recherche, a souscrit un contrat d'assurance en responsabilité civile auprès de SHAM conformément aux dispositions de l'article L1121-10 du code de la santé publique.

Dans le cadre de cette recherche, soumise à la Loi n° 2018-493 du 20 juin 2018 relative à la protection des données personnelles, les données enregistrées à l'occasion de cette recherche font l'objet d'un traitement informatisé à nom de la structure responsable du traitement des données afin d'analyser les résultats de la recherche au regard des objectifs de cette dernière. Conformément au règlement général sur la protection des données (RGPD 2016/679 du Parlement Européen et du Conseil du 27 avril 2016), les données de santé seront traitées à des fins de recherche scientifique dans le respect des droits fondamentaux et des intérêts de la personne se prêtant à la recherche (Article 9, alinéa i et j).

Le responsable du traitement des données dans le cadre de cette étude est le CHR Metz-Thionville, (Téléphone : 03-87-65-54-00). Le délégué à la protection des données du CHR Metz Thionville est Monsieur Albert CRUMBACH (Téléphone : 03-87-55-37-28).

Les personnes participant à la recherche disposent d'un droit d'accès à leurs données, d'un droit de rectification, d'effacement de leurs données, d'un droit à la limitation du traitement ainsi que d'un droit d'opposition au traitement de leurs données personnelles. Ces droits s'exercent auprès de l'Investigateur qui en informera le promoteur de la recherche dans les meilleurs délais.

Les personnes participant à la recherche disposent également d'un droit de réclamation auprès de l'autorité de contrôle en France, à savoir la Commission nationale de l'informatique et des libertés (CNIL).
Les données personnelles ne seront accessibles qu'aux personnes participant à cette recherche et aux personnes chargées par le promoteur de contrôler la qualité de l'étude. Ces données seront identifiées par un numéro de code comportant un numéro d'inclusion (correspondant à l'ordre chronologique de leur inclusion et au numéro de site) ainsi que la 1ère lettre du nom et la 1ère lettre du prénom. Le cas échéant elles pourront également être transmises aux autorités sanitaires habilitées. Dans tous les cas, elles seront exploitées dans les conditions garantissant leur confidentialité.

Ce traitement des données entre dans le cadre de la méthodologie de référence MR001 pour laquelle le CHR Metz-Thionville a signé un engagement de conformité le 05 juillet 2022 (n°2226879 v 0).

- Cette recherche est enregistrée sur le site http://clinicaltrials.gov/ sous le n° NCT05475574.

AMENDEMENT AU PROTOCOLE

Toute modification substantielle, c'est à dire toute modification de nature à avoir un impact significatif sur la protection des personnes, sur les conditions de validité et sur les résultats de la recherche, sur la qualité et la sécurité des produits expérimentés, sur l'interprétation des documents scientifiques qui viennent appuyer le déroulement de la recherche ou sur les modalités de conduite de celle-ci, fait l'objet d'un amendement écrit qui est soumis au promoteur ; celui-ci doit obtenir, préalablement à sa mise en œuvre, un avis favorable du CPP et en informer l'ANSM.

Les modifications non substantielles, c'est à dire celles n'ayant pas d'impact significatif sur quelque aspect de la recherche que ce soit, sont communiquées au CPP à titre d'information.

Tous les amendements sont validés par le promoteur, et par tous les intervenants de la recherche concernés par la modification, avant soumission au CPP.
Tous les amendements au protocole doivent être portés à la connaissance de tous les investigateurs qui participent à la recherche. Les investigateurs s'engagent à en respecter le contenu.

Tout amendement qui modifie la prise en charge des patients ou les bénéfices, risques et contraintes de la recherche fait l'objet d'une nouvelle note d'information et d'un nouveau formulaire de consentement dont le recueil suit la même procédure que celle précitée.

16 CONSERVATION DES DOCUMENTS ET DES DONNEES RELATIVES A LA RECHERCHE

Les documents suivants relatifs à cette recherche sont archivés conformément aux Bonnes Pratiques Cliniques :

– Par les médecins investigateurs :

***- pour une durée de 10 ans suivant la fin de la recherche** (recherches portant sur des produits cosmétiques),*
***- pour une durée de 15 ans suivant la fin de la recherche** (recherches portant sur des médicaments, des dispositifs médicaux ou des dispositifs médicaux de diagnostic in vitro ou recherches ne portant pas sur un produit mentionné à l'article L.5311-1 du code de la santé publique),*
***- pour une durée de 30 ans suivant la fin de la recherche** (recherches portant sur des produits sanguins labiles, des organes, des tissus d'origine humaine ou animale ou des préparations de thérapie cellulaire),*
***- pour une durée de 40 ans suivant la fin de la recherche** (recherches portant sur des médicaments dérivés du sang ou des dispositifs médicaux incorporant une substance qui est susceptible d'être considérée comme un médicament dérivé du sang),*

- Le protocole et les amendements éventuels au protocole
- Les cahiers d'observation (copies)
- Les dossiers source des participants ayant signé un consentement
- Tous les autres documents et courriers relatifs à la recherche

- pour une durée de 30 ans suivant la fin de la recherche
- L'exemplaire original des consentements éclairés signés des participants

Tous ces documents sont sous la responsabilité de l'investigateur pendant la durée réglementaire d'archivage.

– Par le promoteur :

***- pour une durée de 10 ans suivant la fin de la recherche** (recherches portant sur des produits cosmétiques),*
***- pour une durée de 15 ans suivant la fin de la recherche** (recherches portant sur des médicaments, des dispositifs médicaux ou des dispositifs médicaux de diagnostic in vitro ou recherches ne portant pas sur un produit mentionné à l'article L.5311-1 du code de la santé publique),*
***- pour une durée de 30 ans suivant la fin de la recherche** (recherches portant sur des produits sanguins labiles, des organes, des tissus d'origine humaine ou animale ou des préparations de thérapie cellulaire),*
***- pour une durée de 40 ans suivant la fin de la recherche** (recherches portant sur des médicaments dérivés du sang ou des dispositifs médicaux incorporant une substance qui est susceptible d'être considérée comme un médicament dérivé du sang),*

- Le protocole et les amendements éventuels au protocole
- L'original des cahiers d'observation
- Tous les autres documents et courriers relatifs à la recherche

- pour une durée de 30 ans suivant la fin de la recherche
- Un exemplaire des consentements éclairés signés des participants
- Les documents relatifs aux événements indésirables graves

Tous ces documents sont sous la responsabilité du promoteur pendant la durée réglementaire d'archivage.

Aucun déplacement ou destruction ne pourra être effectué sans l'accord du promoteur. Au terme de la durée réglementaire d'archivage, le promoteur sera consulté pour destruction. Toutes les données, tous les documents et rapports pourront faire l'objet d'audit ou d'inspection.

17 REGLES RELATIVES A LA PUBLICATION

17.1 COMMUNICATIONS SCIENTIFIQUES

L'analyse des données fournies par les sites investigateurs est réalisée par la Plateforme d'Appui à la Recherche Clinique du CHR Metz-Thionville. Cette analyse donne lieu à un rapport écrit qui est soumis au promoteur, qui transmettra au Comité de Protection des Personnes et à l'autorité compétente.

Toute communication écrite ou orale des résultats de la recherche doit recevoir l'accord préalable de l'investigateur coordonnateur et, le cas échéant, de tout comité constitué pour la recherche.

La publication des résultats principaux mentionne le nom du promoteur, de tous les investigateurs ayant inclus ou suivi des patients dans la recherche, des méthodologistes, biostatisticiens et data managers ayant participé à la recherche, des membres du(des) comité(s) constitué(s) pour la recherche et la(les) source(s) de financement. Il sera tenu compte des règles internationales d'écriture et de publication (*The Uniform Requirements for Manuscripts* du ICMJE, avril 2010).

Le CHR de Metz-Thionville est propriétaire des données et aucune utilisation ou transmission à un tiers ne peut être effectuée sans son accord préalable.

Le CHR de Metz-Thionville doit être mentionné comme étant le promoteur de la recherche biomédicale et comme soutien financier le cas échéant.

17.2 COMMUNICATION DES RESULTATS AUX PATIENTS

Conformément à la loi n°2002-303 du 4 mars 2002, les patients sont informés, à leur demande, des résultats globaux de la recherche.

17.3 CESSION DES DONNEES

Le recueil et la gestion des données sont assurés par la Plateforme d'Appui à la Recherche Clinique du CHR Metz-Thionville. Les conditions de cession de tout ou partie de la base de données de la recherche sont décidées par le promoteur de la recherche et font l'objet d'un contrat écrit.

18 REFERENCES BIBLIOGRAPHIQUES

1. Ministère de l'emploi et de la solidarité. Secrétariat d'Etat à la santé et à l'action sociale. Maîtrise de la diffusion des bactéries multirésistantes aux antibiotiques - Recommandations pour les établissements de santé. Paris; 1999.

2. Siegel JD, Rhinehart E, Jackson M, Chiarello L, Healthcare Infection Control Practices Advisory Committee. Management of multidrug-resistant organisms in health care settings, 2006. Am J Infect Control. déc 2007;35(10 Suppl 2):S165-193.

3. Organisation mondiale de la santé. Résistance aux antimicrobiens. Aide-mémoire N°194 [Internet]. 2015 [cité 15 sept 2016]. Disponible sur: http://www.who.int/mediacentre/factsheets/fs194/fr/

4. SPF. Bactéries multirésistantes en établissements de santé en 2018 Mission nationale Spares, novembre 2019. Données 2018 du réseau BMR-Raisin [Internet]. [cité 10 sept 2020]. Disponible sur: /import/bacteries-multiresistantes-en-etablissements-de-sante-en-2018-mission-nationale-spares-novembre-2019.-donnees-2018-du-reseau-bmr-raisin

5. Cantón R, Novais A, Valverde A, Machado E, Peixe L, Baquero F, et al. Prevalence and spread of extended-spectrum beta-lactamase-producing Enterobacteriaceae in Europe. Clin Microbiol Infect Off Publ Eur Soc Clin Microbiol Infect Dis. janv 2008;14 Suppl 1:144-53.

6. Lucet JC, Decré D, Fichelle A, Joly-Guillou ML, Pernet M, Deblangy C, et al. Control of a prolonged outbreak of extended-spectrum beta-lactamase-producing enterobacteriaceae in a university hospital. Clin Infect Dis Off Publ Infect Dis Soc Am. déc 1999;29(6):1411-8.

7. Annual surveillance reports on antimicrobial resistance [Internet]. European Centre for Disease Prevention and Control. [cité 10 sept 2020]. Disponible sur: https://www.ecdc.europa.eu/en/antimicrobial-resistance/surveillance-and-disease-data/report

8. Instruction DGOS/PF2/DGS/RI1/DGCS/2015/ 202 du 15 juin 2015 relative au programme national d'actions de prévention des infections associées aux soins (PROPIAS). 2015 [Internet]. [cité 13 sept 2020]. Disponible sur: https://solidarites-sante.gouv.fr/fichiers/bo/2015/15-07/ste_20150007_0000_0034.pdf

9. ANSES_ANSM_SPF_Consommation d'antibiotiques et resistance aux antibiotiques en France. Bilan des surveillance Novembre 2016 [Internet]. [cité 13 sept 2020]. Disponible sur: https://www.anses.fr/fr/system/files/Plaquette_consommation_antibiotiques_resistance_antibiotiques_France2016.pdf

10. HCSP. Prévenir l'émergence des entérobactéries BLSE et lutter contre leur dissémination [Internet]. Rapport de l'HCSP. Paris: Haut Conseil de la Santé Publique; 2010 févr [cité 9 sept 2020]. Disponible sur: https://www.hcsp.fr/explore.cgi/avisrapportsdomaine?clefr=162

11. SF2H_précautions standard_2017 [Internet]. [cité 3 sept 2020]. Disponible sur: https://sf2h.net/wp-content/uploads/2017/06/HY_XXV_PS_versionSF2H.pdf

12. Recommandations nationales - Prévention de la transmission croisée : précautions complémentaires contact - Consensus formalisé d'experts - 2009. :60.

13. HCSP. Actualisation des recommandations relatives aux BHRe [Internet]. Rapport de l'HCSP. Paris: Haut Conseil de la Santé Publique; 2019 déc [cité 2 sept 2020]. Disponible sur: https://www.hcsp.fr/explore.cgi/avisrapportsdomaine?clefr=758

14. Grayson ML, Stewardson AJ, Russo PL, Ryan KE, Olsen KL, Havers SM, et al. Effects of the Australian National Hand Hygiene Initiative after 8 years on infection control practices, health-care worker education, and clinical outcomes: a longitudinal study. Lancet Infect Dis. 2018;18(11):1269-77.

15. Pittet D, Hugonnet S, Harbarth S, Mourouga P, Sauvan V, Touveneau S, et al. Effectiveness of a hospital-wide programme to improve compliance with hand hygiene. Infection Control Programme. Lancet Lond Engl. 14 oct 2000;356(9238):1307-12.

16. Boyce JM, Pittet D, Healthcare Infection Control Practices Advisory Committee. Society for Healthcare Epidemiology of America. Association for Professionals in Infection Control. Infectious Diseases Society of America. Hand Hygiene Task Force. Guideline for Hand Hygiene in Health-Care Settings: recommendations of the Healthcare Infection Control Practices Advisory Committee and the HICPAC/SHEA/APIC/IDSA Hand Hygiene Task Force. Infect Control Hosp Epidemiol. déc 2002;23(12 Suppl):S3-40.

17. Clarivet B, Grau D, Jumas-Bilak E, Jean-Pierre H, Pantel A, Parer S, et al. Persisting transmission of carbapenemase-producing Klebsiella pneumoniae due to an environmental reservoir in a university hospital, France, 2012 to 2014. Euro Surveill Bull Eur Sur Mal Transm Eur Commun Dis Bull. 28 avr 2016;21(17).

18. Anderson DJ, Chen LF, Weber DJ, Moehring RW, Lewis SS, Triplett PF, et al. Enhanced terminal room disinfection and acquisition and infection caused by multidrug-resistant organisms and Clostridium difficile (the Benefits of Enhanced Terminal Room Disinfection study): a cluster-randomised, multicentre, crossover study. Lancet Lond Engl. 25 2017;389(10071):805-14.

19. Marra AR, Edmond MB, Schweizer ML, Ryan GW, Diekema DJ. Discontinuing contact precautions for multidrug-resistant organisms: A systematic literature review and meta-analysis. Am J Infect Control. 2018;46(3):333-40.

20. Shardell M, Harris AD, El-Kamary SS, Furuno JP, Miller RR, Perencevich EN. Statistical analysis and application of quasi experiments to antimicrobial resistance intervention studies. Clin Infect Dis Off Publ Infect Dis Soc Am. 1 oct 2007;45(7):901-7.

21. Morgan DJ, Murthy R, Munoz-Price LS, Barnden M, Camins BC, Johnston BL, et al. Reconsidering contact precautions for endemic methicillin-resistant Staphylococcus aureus and vancomycin-resistant Enterococcus. Infect Control Hosp Epidemiol. oct 2015;36(10):1163-72.

22. Girou E, Chai SHT, Oppein F, Legrand P, Ducellier D, Cizeau F, et al. Misuse of gloves: the foundation for poor compliance with hand hygiene and potential for microbial transmission? J Hosp Infect. juin 2004;57(2):162-9.

23. Jolivet S, Vaillant L, Poncin T, Lolom I, Gaudonnet Y, Rondinaud E, et al. Prevalence of carriage of extended-spectrum β-lactamase-producing enterobacteria and associated factors in a French hospital. Clin Microbiol Infect Off Publ Eur Soc Clin Microbiol Infect Dis. déc 2018;24(12):1311-4.

24. Dhar S, Marchaim D, Tansek R, Chopra T, Yousuf A, Bhargava A, et al. Contact precautions: more is not necessarily better. Infect Control Hosp Epidemiol. mars 2014;35(3):213-21.

25. Renaudin L, Llorens M, Goetz C, Gette S, Citro V, Poulain S, et al. Impact of Discontinuing Contact Precautions for MRSA and ESBLE in an Intensive Care Unit: A Prospective Noninferiority Before and After Study. Infect Control Hosp Epidemiol. 2017;38(11):1342-50.

26. Zahar J-R, Poirel L, Dupont C, Fortineau N, Nassif X, Nordmann P. About the usefulness of contact precautions for carriers of extended-spectrum beta-lactamase-producing Escherichia coli. BMC Infect Dis. 12 nov 2015;15:512.

27. Tschudin-Sutter S, Frei R, Schwahn F, Tomic M, Conzelmann M, Stranden A, et al. Prospective Validation of Cessation of Contact Precautions for Extended-Spectrum β-Lactamase-Producing Escherichia coli(1). Emerg Infect Dis. 2016;22(6):1094-7.

28. Kluytmans-van den Bergh MFQ, Bruijning-Verhagen PCJ, Vandenbroucke-Grauls CMJE, de Brauwer EIGB, Buiting AGM, Diederen BM, et al. Contact precautions in single-bed or multiple-bed rooms for patients with extended-spectrum β-lactamase-producing Enterobacteriaceae in Dutch hospitals: a cluster-randomised, crossover, non-inferiority study. Lancet Infect Dis. 2019;19(10):1069-79.

29. Maechler F, Schwab F, Hansen S, Fankhauser C, Harbarth S, Huttner BD, et al. Contact isolation versus standard precautions to decrease acquisition of extended-spectrum β-lactamase-producing Enterobacterales in non-critical care wards: a cluster-randomised crossover trial. Lancet Infect Dis. 2020;20(5):575-84.

30. Denkel LA, Maechler F, Schwab F, Kola A, Weber A, Gastmeier P, et al. Infections caused by extended-spectrum β-lactamase-producing Enterobacterales after rectal colonization with ESBL-producing Escherichia coli or Klebsiella pneumoniae. Clin Microbiol Infect Off Publ Eur Soc Clin Microbiol Infect Dis. août 2020;26(8):1046-51.

31. Zahar JR, Garrouste-Orgeas M, Vesin A, Schwebel C, Bonadona A, Philippart F, et al. Impact of contact isolation for multidrug-resistant organisms on the occurrence of medical errors and adverse events. Intensive Care Med. déc 2013;39(12):2153-60.

32. Harris AD, Pineles L, Belton B, Johnson JK, Shardell M, Loeb M, et al. Universal glove and gown use and acquisition of antibiotic-resistant bacteria in the ICU: a randomized trial. JAMA. 16 oct 2013;310(15):1571-80.

33. Cooper BS, Stone SP, Kibbler CC, Cookson BD, Roberts JA, Medley GF, et al. Isolation measures in the hospital management of methicillin resistant Staphylococcus aureus (MRSA): systematic review of the literature. BMJ. 4 sept 2004;329(7465):533.

34. Gruber I, Heudorf U, Werner G, Pfeifer Y, Imirzalioglu C, Ackermann H, et al. Multidrug-resistant bacteria in geriatric clinics, nursing homes, and ambulant care--prevalence and risk factors. Int J Med Microbiol IJMM. déc 2013;303(8):405-9.

35. Denkinger CM, Grant AD, Denkinger M, Gautam S, D'Agata EMC. Increased multi-drug resistance among the elderly on admission to the hospital--a 12-year surveillance study. Arch Gerontol Geriatr. févr 2013;56(1):227-30.

36. McElhaney JE, Effros RB. Immunosenescence: what does it mean to health outcomes in older adults? Curr Opin Immunol. août 2009;21(4):418-24.

37. Pilmis B, Cattoir V, Lecointe D, Limelette A, Grall I, Mizrahi A, et al. Carriage of ESBL-producing Enterobacteriaceae in French hospitals: the PORTABLSE study. J Hosp Infect. mars 2018;98(3):247-52.

38. rapport_claris_version_finale.pdf [Internet]. [cité 14 sept 2020]. Disponible sur: https://solidarites-sante.gouv.fr/IMG/pdf/rapport_claris_version_finale.pdf

39. Hugonnet S, Harbarth S, Sax H, Duncan RA, Pittet D. Nursing resources: a major determinant of nosocomial infection? Curr Opin Infect Dis. août 2004;17(4):329-33.

40. Needleman J, Buerhaus P, Pankratz VS, Leibson CL, Stevens SR, Harris M. Nurse staffing and inpatient hospital mortality. N Engl J Med. 17 mars 2011;364(11):1037-45.

41. Carey D, Price K, Neal S, Compton C, Ash C, Bryan N, et al. The impact of discontinuing contact precautions for multidrug resistant organisms at a less than 400-bed level II teaching hospital and a community hospital: A 3-month pilot study. Am J Infect Control. 2020;48(3):333-6.

42. SF2H_recommandations_air-ou-gouttelettes_2013.pdf [Internet]. [cité 5 sept 2020]. Disponible sur: https://www.sf2h.net/wp-content/uploads/2013/03/SF2H_recommandations_air-ou-gouttelettes_2013.pdf

43. Sax H, Allegranzi B, Chraïti M-N, Boyce J, Larson E, Pittet D. The World Health Organization hand hygiene observation method. Am J Infect Control. déc 2009;37(10):827-34.

44. Mitchell R, Roth V, Gravel D, Astrakianakis G, Bryce E, Forgie S, et al. Are health care workers protected? An observational study of selection and removal of personal protective equipment in Canadian acute care hospitals. Am J Infect Control. mars 2013;41(3):240-4.

45. Zigmond A.S., Snaith R.P. The Hospital Anxiety and Depression Scale. Acta Psychiatr. Scand., 1983, 67, 361-370

46. Bonten MJ, Slaughter S, Ambergen AW, Hayden MK, van Voorhis J, Nathan C, et al. The role of « colonization pressure » in the spread of vancomycin-resistant enterococci: an important infection control variable. Arch Intern Med. 25 mai 1998;158(10):1127-32.

47. Ajao AO, Johnson JK, Harris AD, Zhan M, McGregor JC, Thom KA, et al. Risk of acquiring extended-spectrum β-lactamase-producing Klebsiella species and Escherichia coli from prior room occupants in the intensive care unit. Infect Control Hosp Epidemiol. mai 2013;34(5):453-8.

48. Kaier K, Meyer E, Dettenkofer M, Frank U. Epidemiology meets econometrics: using time-series analysis to observe the impact of bed occupancy rates on the spread of multidrug-resistant bacteria. J Hosp Infect. oct 2010;76(2):108-13.

49. Jolivet S, Lolom I, Bailly S, Bouadma L, Lortat-Jacob B, Montravers P, et al. Impact of colonization pressure on acquisition of extended-spectrum β-lactamase-producing Enterobacterales and meticillin-resistant Staphylococcus aureus in two intensive care units: a 19-year retrospective surveillance. J Hosp Infect. mai 2020;105(1):10-6.

50. Torres-Gonzalez P, Cervera-Hernandez ME, Niembro-Ortega MD, Leal-Vega F, Cruz-Hervert LP, García-García L, et al. Factors Associated to Prevalence and Incidence of Carbapenem-Resistant Enterobacteriaceae Fecal Carriage: A Cohort Study in a Mexican Tertiary Care Hospital. PloS One. 2015;10(10):e0139883.

51. Schoevaerdts D, Bogaerts P, Grimmelprez A, de Saint-Hubert M, Delaere B, Jamart J, et al. Clinical profiles of patients colonized or infected with extended-spectrum beta-lactamase producing Enterobacteriaceae isolates: a 20 month retrospective study at a Belgian University Hospital. BMC Infect Dis. 12 janv 2011;11:12.

52. Schoevaerdts D, Verroken A, Huang T-D, Frennet M, Berhin C, Jamart J, et al. Multidrug-resistant bacteria colonization amongst patients newly admitted to a geriatric unit: a prospective cohort study. J Infect. août 2012;65(2):109-18.

53. Neumann N, Mischler D, Cuny C, Hogardt M, Kempf VAJ, Heudorf U. [Multidrug-resistant organisms (MDRO) in patients in outpatient care in the Rhine-Main region, Germany, in 2014: Prevalence and risk factors]. Bundesgesundheitsblatt Gesundheitsforschung Gesundheitsschutz. févr 2016;59(2):292-300.

54. Schuirmann DJ. A comparison of the two one-sided tests procedure and the power approach for assessing the equivalence of average bioavailability. J Pharmacokinet Biopharm. déc 1987;15(6):657-80.

55. Elsevier, N A-C. Gériatrie [Internet]. Elsevier Connect. [cité 9 mars 2022]. Disponible sur: https://www.elsevier.com/fr-fr/connect/medecine-generale/geriatrie

56. Hemming K, Taljaard M. Sample size calculations for stepped wedge and cluster randomised trials: a unified approach. J Clin Epidemiol 2016;69:137-46.

57. Quan H, Li B, Couris CM, Fushimi K, Graham P, Hider P, et al. Updating and validating the Charlson comorbidity index and score for risk adjustment in hospital discharge abstracts using data from 6 countries. Am J Epidemiol 2011;173(6):676-82.

ANNEXES

ANNEXE 1. GRILLE D'AUDIT PULPE' FRICTION

Identification des freins à la friction hydroalcoolique auprès des soignants

PULPE' FRICTION

RéPias MATIS Réseau de Prévention des Infections Associées aux Soins

Fiche médical/ paramédical

Fiche à compléter pour chaque professionnel de santé.

Concernant les éléments de langage et modalités d'introduction de cette enquête auprès des professionnels, consulter la rubrique « **recette pour communiquer avec les professionnels** » de la méthodologie.

N° de fiche (sera auto-incrémentée lors de la saisie) ....

1) Etes-vous un professionnel médical/ paramédical ?

....

2) Je vous demande de penser aux actes de soin que vous avez fait ces derniers jours. Vous être plutôt (cochez un chiffre):

Toujours lavage au savon — Autant lavage que friction — Toujours friction

0	1	2	3	4	5	6	7	8	9	10

3) Je vous demande de continuer à penser aux actes de soin que vous avez fait ces derniers jours, et pour chacune des situations, de m'indiquer, sur l'échelle allant de 0 (jamais) à 10 (toujours), à quelle fréquence vous avez fait une FRICTION des mains.

3a) Après être rentré dans la chambre et avant de toucher le patient/résident (par exemple pour l'examiner, ou lui faire un soin non invasif) *(Curseur à positionner sur une ligne figurant 0 à 10)*

Jamais — Toujours

0	1	2	3	4	5	6	7	8	9	10

Quelle est l'importance pour vous de faire une HYGIENE DES MAINS avant de toucher un patient/résident ?

Pas du tout important — Le plus important

0	1	2	3	4	5	6	7	8	9	10

3b) Juste avant de faire un geste invasif (injection, cathéter, incision, sondage, intubation…) *(Curseur à positionner sur une ligne figurant 0 à 10 ou non concerné)*

Jamais — Toujours

0	1	2	3	4	5	6	7	8	9	10

NC

Quelle est l'importance pour vous de faire une HYGIENE DES MAINS avant un geste invasif ?

Pas du tout important — Le plus important

0	1	2	3	4	5	6	7	8	9	10

3c) Après le dernier contact physique avec un patient et avant de sortir de la chambre ?

Jamais — Toujours

0	1	2	3	4	5	6	7	8	9	10

Quelle est l'importance pour vous de l'hygiène des mains après le dernier contact avec le patient/résident ?

Pas du tout important — Le plus important

0	1	2	3	4	5	6	7	8	9	10

3d) Après avoir touché l'environnement proche du patient et avant de sortir de la chambre ? (par exemple son lit, sa perfusion, le scope, les machines proches, la seringue électrique…)

Jamais — Toujours

0	1	2	3	4	5	6	7	8	9	10

Quelle est l'importance pour vous de faire une HYGIENE DES MAINS après avoir touché l'environnement proche du patient/résident ?

Pas du tout important — Le plus important

0	1	2	3	4	5	6	7	8	9	10

4) Quelles sont les raisons principales qui peuvent ou qui pourraient vous empêcher de faire une FRICTION des mains ? *(2 choix minimum, 3 maximum)*

- ☐ Manque d'information
- ☐ Pas concerné
- ☐ Geste professionnel non à risque
- ☐ Pas convaincu de l'intérêt
- ☐ Pas une priorité
- ☐ Pas l'habitude
- ☐ Manque de temps
- ☐ Ne sait pas comment faire (technique)
- ☐ Nocivité du produit
- ☐ Produit inconfortable ou désagréable
- ☐ Coût du produit
- ☐ Disponibilité du produit
- ☐ Allergie avérée

ANNEXE 2. MANUEL DE LABORATOIRE

Etude Ger-SP

Impact de l'abandon des précautions complémentaires contact au profit des précautions « standard » sur la transmission des entérobactéries productrices de β-lactamases à spectre étendu en gériatrie aigue

CODE PROMOTEUR : 2021-05-CHRMT

PROTOCOLE DE RECHERCHE IMPLIQUANT LA PERSONNE HUMAINE

NE COMPORTANT QUE DES RISQUES ET DES CONTRAINTES MINIMES

(2° DE L'ARTICLE L.1121-1 DU CODE DE LA SANTE PUBLIQUE)

Version 1.2 du 18/11/2022

N° IDRCB ANSM : 2021-A02951-40

Avis favorable du CPP Ouest II le 31/08/2022

Information ANSM le 31/08/2022

PROMOTEUR

CHR METZ-THIONVILLE – Hôpital de Mercy

1 Allée du Château - CS 45001, 57085 METZ Cedex 03

INVESTIGATEUR COORDONNATEUR

Dr. Noël BLETTNER
Service de gériatrie
CHR Metz-Thionville, Hôpital de Mercy
: 1 allée du Château - CS 45001 - 57085 Metz Cedex 03
: 03 87 55 78 46
: n.blettner@chr-metz-thionville.fr

COORDONNATEUR SCIENTIFIQUE

Dr Laurie RENAUDIN
Service d'hygiène hospitalière
CHR Metz-Thionville, Hôpital de Mercy
: 1, allée du Château - CS 45001 - 57 085 Metz Cedex 03
: 03 87 55 30 25
: l.renaudin@chr-metz-thionville.fr

1. CONTACTS

Rôle	NOM Prénom	Structure	Coordonnées (tél/courriel)
Biologiste	Dr PEREZ Pascale	Service Laboratoire<br>CHR Metz-Thionville, Hôpital de Mercy<br>🖃 : 1 allée du Château - CS 45001 – 57 085 Metz Cedex 03	✆ : 03 87 55 36 85<br>✉ : p.perez@chr-metz-thionville.fr
Coordinateur scientifique	Dr RENAUDIN Laurie	Service d'Hygiène Hospitalière<br>CHR Metz-Thionville, Hôpital de Mercy<br>🖃 : 1 allée du Château - CS 45001 – 57 085 Metz Cedex 03	✆ : 03 87 55 30 25<br>✉ : l.renaudin@chr-metz-thionville.fr
Investigateur Principal Centre N°2/ Coordonnateur : Hôpital de Mercy	Dr BLETTNER Noël	Service de gériatrie<br>CHR Metz-Thionville, Hôpital de Mercy<br>🖃 : 1 allée du Château - CS 45001 – 57 085 Metz Cedex 03	✆ : 03 87 55 38 86<br>✉ : n.blettner@chr-metz-thionville.fr
Investigateur Principal Centre N°1 : Hôpital Bel Air	Dr AZZEMOU Azzeddine	Service de gériatrie<br>CHR Metz-Thionville, Hôpital Bel Air<br>🖃 : 1-3 rue de Friscaty<br>57126 Thionville Cedex	✆ : 03 82 55 87 10<br>✉ : a.azzemou@chr-metz-thionville.fr
Technicienne d'étude clinique Centre N°1 : Hôpital Bel Air	URBING Arielle	Service Plateforme d'Appui à la Recherche Clinique (PARC)<br>CHR Metz-Thionville, Hôpital Bel Air<br>🖃 : 1-3 rue de Friscaty<br>57126 Thionville Cedex	✆ : 03 82 55 89 06<br>✉ : a.urbing@chr-metz-thionville.fr
Cheffe de projet	EL NAR Arpiné	Service Plateforme d'Appui à la Recherche Clinique (PARC)<br>CHR Metz-Thionville, Hôpital de Mercy<br>🖃 : 1 allée du Château - CS 45001 – 57 085 Metz Cedex 03	✆ : 03 87 55 77 66<br>✉ : a.elnar@chr-metz-thionville.fr
Technicienne d'étude clinique Centre N°2 : Hôpital de Mercy	JUNKE Mélanie		✆ : 03 87 17 98 86<br>✉ : m.junke@chr-metz-thionville.fr
Destinataire échantillons typage moléculaire	BERTRAND Xavier	Service d'hygiène hospitalière<br>Bâtiment PC Bio +3<br>Centre Hospitalier Régional Universitaire Hôpital Jean Minjoz (Besançon)<br>🖃 : 3 boulevard Fleming – 25030 Besançon	✆ : 06 89 72 20 03<br>✉ : xbertrand@chu-besancon.fr ;<br>✉ : xavier.bertrand@univ-fcomté.fr

2. KITS

Les prélèvements rectaux seront collectés sur le support eSwab™ – Rose (COPAN) . Ce kit contient un écouvillon et un milieu de conservation (figure ci-dessous).

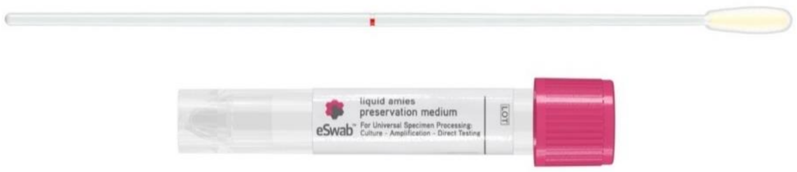

Figure 1 : Ecouvillon de microbiologie eSwab Rose de COPAN

3. CALENDRIER DES PRELEVEMENTS RECTAUX

Dans le cadre de la recherche, les prélèvements rectaux seront réalisés :

- **Dans les 48h après l'admission du patient en hospitalisation dans le service de gériatrie**
- **A la sortie du patient**

4. PROCEDURE DES PRELEVEMENTS RECTAUX

Matériel nécessaire :

- Gants à usage unique non stériles
- 1 kit de microbiologie Eswab Rose (COPAN)
- Dosette de sérum physiologique stérile
- Solution hydro alcoolique (SHA)

Technique

Préalables :

- Information du patient et/ou des représentants légaux si le patient est incapable d'exprimer son consentement. Il faut expliquer au patient le geste et l'objectif du prélèvement de manière loyale et claire.

Modalités de prélèvement :

Prélèvement à réaliser

↓

Effectuer une friction avec SHA

↓

Mettre les gants à usage unique

↓

Introduire l'écouvillon au niveau intrarectal profond (cf. prise de température) et recueillir des matières fécales (visibles à l'œil nu) **ou**
Tremper l'écouvillon dans les selles recueillies dans un pot à coproculture

↓

Placer l'écouvillon dans le milieu de conservation Eswab Rose (Copan)
Capuchonner le tube

↓

Enlever les gants

↓

Effectuer une friction avec SHA

↓

Etiqueter le prélèvement, remplir la demande de prélèvement (cf. Manuel de prélèvement), tracer

↓

Prélèvement réalisé

5. CIRCUIT D'ANALYSE ET D'ENVOI DES PRELEVEMENTS

5.1. Prélèvements rectaux de dépistage, circuit service de gériatrie – laboratoire de microbiologie

Afin de respecter l'insu, un circuit est organisé avec le laboratoire de microbiologique du CHR et les résultats ne sont pas rendus sur le dossier patient informatisé. A savoir, l'étude propose un schéma en double aveugle, ce qui implique que les résultats de dépistage rectaux réalisés ne seront pas communiqués aux patients ni aux personnels du service de gériatrie. Ils seront tracés dans le CRF de l'étude.

Le circuit sera organisé comme suit :

Quoi	Qui	Comment
**1. Prescription d'une ordonnance de prélèvement rectal à réaliser dans les 48h après l'admission / ou à la sortie**	Médecin gériatre	Cf. ordonnance type (Annexe 6)<br><br>L'identification du patient sera réalisée de la façon suivante :<br>Coller une étiquette patient<br>ou<br>Compléter les données NOM/Prénom et DDN<br>Le prescripteur inscrit sur l'ordonnance sera le service de gériatrie
**2. Réalisation du prélèvement rectal + étiquetage du prélèvement**	IDE du service de gériatrie	Le prélèvement est effectué par l'IDE du service.<br>L'identification du prélèvement sera réalisée par l'IDE service de la façon suivante :<br>Coller une étiquette patient<br>ou<br>Compléter les données NOM/Prénom et DDN<br><br>Le prélèvement est stocké au réfrigérateur à 4°C dans l'attente de l'envoi par la TEC de la PARC
**3. Anonymisation du prélèvement rectal et de l'ordonnance**	TEC PARC	Le prélèvement rectal et l'ordonnance seront anonymisés par la TEC en apposant une étiquette spécifique (annexe 7) :<br><br>**Nom = GERSP-InitialesNomPrénom**<br>**Prénom = N°centre-N°inclusion**<br>**DDN = date de naissance du patient (mois/année)**<br>**Date de prélèvement = jour/mois/année**
**4. Envoi du prélèvement au laboratoire de microbiologie**	TEC PARC	Par le circuit habituel (les échantillons peuvent être conservés à 4°C pendant 24 H avant d'être acheminés à température ambiante à l'hôpital de Mercy pour analyse, accompagnés de l'ordonnance et de la fiche N°1)
**5. Réception du prélèvement et analyse**	Personnels du laboratoire de microbiologie	Le laboratoire devrait réceptionner l'échantillon d'écouvillon rectal accompagné de l'ordonnance et de la fiche de prélèvement de l'échantillon (fiche N°1).<br><br>Si des échantillons sont manquants et/ou les documents non complétées, contacter immédiatement la technicienne d'étude clinique (TEC) en charge de l'étude.<br><br>Le technicien qui prend en charge les prélèvements renseigne les informations décrites dans la fiche (partie LABO), notamment la date et l'heure de l'analyse.<br><br>Le prélèvement sera ensuite enregistré dans le système d'information du laboratoire (logiciel GLIMS).<br>Les résultats ne seront pas disponibles sur le dossier patient informatisé (logiciel DxCare) car l'identification du patient (cf. point 1 et 2 ci-dessus) ne permettra pas de faire le lien avec son dossier.<br>Les résultats seront envoyés par courrier interne au service d'hygiène hospitalière et à la plateforme d'appui à la recherche clinique<br><br>***Coordonnées :***<br>*Service Plateforme d'Appui à la Recherche Clinique*

	Hôpital de Mercy ; CHR Metz-Thionville,<br>*1 allée du Château - CS 45001*<br>*57 085 Metz Cedex 03*<br>*Tél : 03 87 17 98 82*<br>*Courriel : recherche-investigation@chr-metz-thionville.fr)*<br><br>Ils seront ensuite tracés dans le CRF de l'étude.<br>Les souches EBLSE positives seront conservées dans des cryobilles par le laboratoire et conservées à -80°C dans un espace commun LABO/PARC.

NB : L'insu est levé en cas de découverte fortuite de BHRe (= bactérie hautement résistante, différente des EBLSE, recherchée sur des dépistages rectaux), c'est-à-dire lorsqu'une BHRe est identifiée sur un dépistage rectal chez un patient non connu porteur. En effet, cette situation nécessite la mise en place de mesures spécifiques de prévention de transmission (PCC pour le patient porteur et le dépistage des patients contact) et un signalement aux tutelles (bénéfice individuel).

5.2. Prélèvements cliniques, circuit service de gériatrie – laboratoire de microbiologie

Des prélèvements microbiologiques cliniques seront uniquement réalisés en cas de suspicion d'infection (urinaire, bactériémie, infection pulmonaire, etc.) selon les pratiques habituelles. Les résultats de ces prélèvements seront communiqués aux équipes comme habituellement. Un prélèvement positif (prélèvement clinique) sera anonymisé et envoyé au service d'hygiène hospitalière du CHU de Besançon de Pr Xavier BERTRAND pour réalisation d'un typage moléculaire (voir partie 5.3 ci-dessous).

5.3. Circuit laboratoire (CHRMT) – PARC (CHRMT) – laboratoire (CHU Besançon)

Tout prélèvement positif (dépistage rectal ou prélèvement clinique réalisé selon la prise en charge habituelle) sera envoyé au service d'hygiène hospitalière du CHU de Besançon de Pr Xavier BERTRAND pour réalisation d'un typage moléculaire (voir schéma récapitulatif Figure 2 ci-dessous).

Quoi	Qui	Comment
**En cas de prélèvements positifs, le laboratoire de microbiologie conserve la souche à -80°C dans un espace commun LABO/PARC**<br><br>*** Dans le cas où un prélèvement microbiologique clinique réalisé dans la pratique habituelle (sang, urine, selles, expectoration, etc…) est positif, le service d'hygiène hospitalière se charge d'informer le laboratoire et la PARC afin de stocker les souches pour envoi ultérieur pour typage moléculaire**	TEC/PARC	Les cryobilles contenant les souches EBLSE-positives sont anonymisées à l'aide d'une contre-étiquette (étapes A7/B5 Figure 2 ci-dessous et annexe 8).<br>Avant l'envoi, la souche EBLSE positive est repiquée sur un milieu gélosé incubé à 37°C pendant 24H puis ensemencée sur un tube gélosé de transport (étapes A9/B7 Figure 2 ci-dessous).<br><br>Une contre-étiquette sera placée le tube de gélose avec les informations concernant l'identification du patient. L'étiquette spécifique au prélèvement ne sera **pas nominative.** L'identification du patient sera réalisée de la façon suivante (annexe 8) :<br><br>**Type de prélèvement : ______________________**<br>**Référence Patient : N°centre-N°inclusion**<br>**Initiales : InitialesNomPrénom**<br>**DDN : date de naissance du patient (mois/année)**<br>**Date de prélèvement : (jour/mois/année)**
**Envoi des souches de façon semestrielle au CHU de Besançon**	TEC/PARC<br>LABO	Les souches positives sont envoyées dans des tubes de gélose à température ambiante.<br>La préparation des échantillons pour envoi est décrite dans la fiche N°2.<br>Les échantillons prêts pour envoi devront être accompagnés par une liste détaillée des échantillons (fiche N°3).<br>La demande d'envoi des échantillons devra se faire auprès du CHR Metz-Thionville via une fiche de demande de transport échantillons (fiche N°4).<br><br>A réception, le CHU de Besançon renvoie au promoteur une fiche d'accusé de réception (fiche N°5)

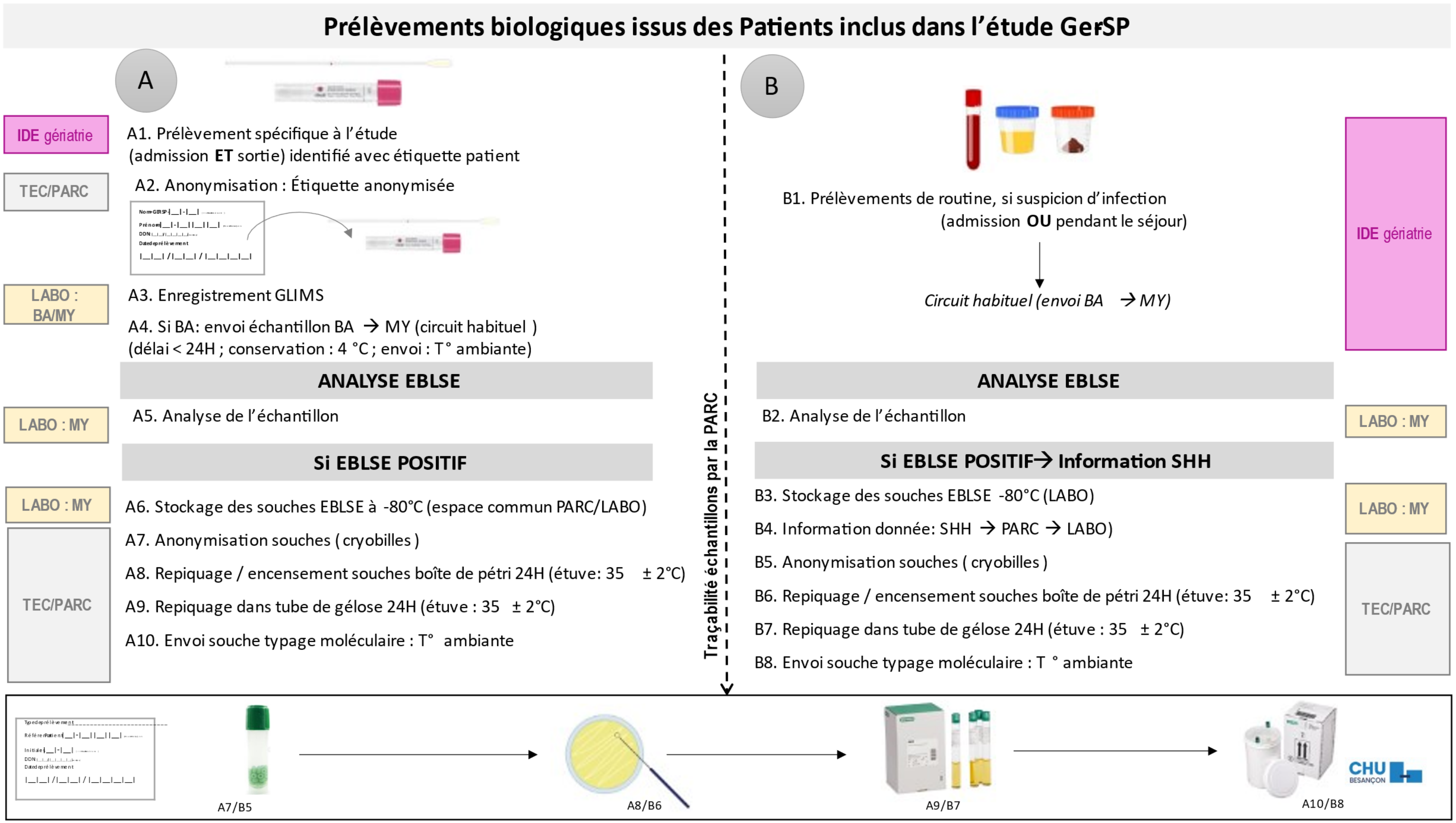

Figure 2 : Schéma récapitulatif des différentes étapes du circuit de prélèvements des échantillons

FICHE N°1 : PRELEVEMENT ECHANTILLON

ETUDE GER-SP

Nom = GERSP- |__|-|__| (Initiales Nom-Prénom)
Prénom = |__|-|__||__||__| (N°centre-N°inclusion)
DDN : |__|__| / |__|__|__|__| (mm/aaaa)
Date de prélèvement :
|__|__| / |__|__| / |__|__|__|__|

INFORMATIONS DU PATIENT		
Référence Patient_Ger-SP	|__|-|__||__||__| (N°centre-N°inclusion)	
Initiales Patient	|__|-|__| (Nom usuel - Prénom)	
Date de naissance	|__|__| / |__|__|__|__| (mm/aaa)	
Date de signature du consentement	|__|__|/|__|__| / |__|__|__|__| (jj/mm/aaa)	
Sexe	☐ Homme	☐ Femme
Visite du patient	☐ Admission d'hospitalisation<br>☐ Sortie d'hospitalisation	

ETAPE 1 : PRELEVEMENTS (à remplir par l'IDE Service gériatrie)	
Prélèvement rectal	☐ 1 écouvillon dans un support eSwabTM Rose (COPAN)
Date et heure du prélèvement rectal	|__|__|/|__|__| / |__|__|__|__| (jj/mm/aaa) à |__|__| : |__|__|
Nom du préleveur	....

☐ Le prélèvement est stocké à 4°C au réfrigérateur dans l'attente de l'envoi par la TEC de la PARC

ETAPE 2 : TRAITEMENTS DES PRELEVEMENTS (à remplir par le laboratoire)	
Prélèvement 1 écouvillon dans un support eSwabTM Rose (COPAN)	
Enregistrement échantillon dans GLIMS (étiquette ci-dessus)	
Date et heure d'analyse	|__|__|/|__|__| / |__|__|__|__| (jj/mm/aaa) à |__|__| : |__|__|

ETAPE 3 : RESULTAT ET STOCKAGE DES ECHANTILLONS (à remplir par la PARC)	
Résultat analyse EBLSE	☐ Positif ☐ Négatif
☞ *Si résultat EBLSE positif*<br>Date et heure de congélation (-80°C)	|__|__|/|__|__| / |__|__|__|__| (jj/mm/aaa) à |__|__| : |__|__|
Stockage : LIEU_N° boite	LABO/PARC_N°..

Coordonnées de la TEC en charge de l'étude :

☞ Nom, Prénom et adresse mail : ....

☏ Numéro de téléphone : ....

FICHE D’ENVOI ECHANTILLONS

ETUDE GER-SP

A joindre aux échantillons lors de leur envoi pour typage moléculaire

**PREPARATION DE L’ENVOI DES ECHANTILLONS (à réaliser par TEC/Promoteur)**	
- Enrober les tubes de géloses dans du papier bulles	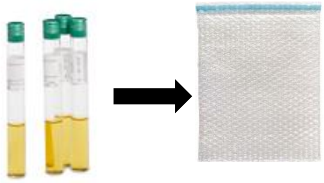
- Ouvrir le carton de transport à rabats (kit de transport de substances biologiques BIORAD fourni par le service LABO du CHR Metz-Thionville)<br><br>- Sortir le pot en plastique cylindrique et retirer la mousse absorbante	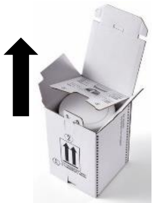
- Insérer les tubes de géloses enrobés dans du papier bulles dans le pot en plastique cylindrique	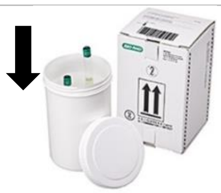
- Placer la boîte contenant les tubes gélosés dans le carton de transport de substances infectieuses.	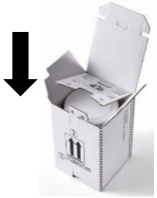
- Faire la demande de transport des échantillons auprès du service Laboratoire de biologie du CHR Metz-Thionville	Fiche de demande de transport

FCHE N°3 : LISTE ENVOI ECHANTILLONS

ETUDE GER-SP

A joindre aux échantillons lors de leur envoi pour typage moléculaire

BOITE N° : ……………………………………………………………………………………………………

LISTE DES ECHANTILLONS A ENVOYER (à remplir par TEC/Promoteur)

Référence Patient (N° centre – N° inclusion)	Type de prélèvement[1]	Date de prélèvement	Visite[2]

[1] Préciser le type de prélèvement parmi les choix suivants : écouvillon rectal, sang, urine, selles, expectoration ou autre : à préciser…

[2] Préciser la visite du prélèvement : à l'admission, pendant le séjour ou à la sortie d'hospitalisation

FICHE N°4 : DEMANDE TRANSPORT ECHANTILLONS

ETUDE GER-SP

A communiquer au LABORATOIRE du CHR Metz-Thionville

DEMANDE DE TRANSPORT ECHANTILLONS (à compléter par la PARC)
- La demande de transport est réalisée via une plateforme dédiée propre au laboratoire du CHR Metz-Thionville.<br>- Date d'envoi des échantillons : |__|__|/|__|__| / |__|__|__|__| (jj/mm/aaa)<br>- Nombre de Boîtes à envoyer : ……………………………………………………………………………………<br>- Numéros de Boîtes : ……………………………………………………………………………………………

DESTINATION DES PRELEVEMENTS (à communiquer au LABORATOIRE)
**Centre destinataire :**<br><br>Service d'hygiène hospitalière<br>Bâtiment PC Bio +3<br>Centre Hospitalier Régional Universitaire Hôpital Jean Minjoz (Besançon)<br>🖃 : 3 boulevard Fleming – 25030 Besançon<br>✆ : 06 89 72 20 03<br>🖂 : xbertrand@chu-besancon.fr ;<br>🖂 : xavier.bertrand@univ-fcomté.fr

FICHE N°5 : ACCUSE RECEPTION ECHANTILLONS

ETUDE GER-SP

RECEPTION ECHANTILLONS (à compléter par CHU BESANÇON)
Nom, Prénom de la personne réceptionnant les échantillons : ……………………………………………………………<br>Nombre de Boîtes reçues : ……………………………………………………………………………………………………<br>Numéros de Boîtes reçues : ……………………………………………………………………………………………………<br>Date de réception : |__|__|/|__|__| / |__|__|__|__| (jj/mm/aaa)
Fiche à renvoyer au CHR Metz-Thionville :<br>☞ Nom, Prénom et adresse mail : ……………………………………………………………………………………………<br>☏ Numéro de téléphone : ……………………………………………………………………………………………………

ANNEXE 3. ECHELLE HAD (HOSPITAL ANXIETY AND DEPRESSION SCALE)

Auto-questionnaire évaluant le niveau de l'anxiété et de la dépression chez les patients

Score	Anxiété	Score	Dépression
	Je me sens tendu ou énervé :		Je prends plaisir aux mêmes choses qu'autrefois
3	❐ la plupart du temps	0	❐ oui, tout autant
2	❐ souvent	1	❐ pas autant
1	❐ de temps en temps	2	❐ un peu seulement
0	❐ jamais	3	❐ presque plus
	J'ai une sensation de peur comme si quelque chose		Je ris facilement et vois le bon côté des choses
	d'horrible allait m'arriver	0	❐ autant que par le passé
3	❐ oui, très nettement	1	❐ plus autant qu'avant
2	❐ oui, mais ce n'est pas grave	2	❐ vraiment moins qu'avant
1	❐ un peu, mais cela ne m'inquiète pas	3	❐ plus du tout
0	❐ pas du tout		
	Je me fais du souci :		Je suis de bonne humeur :
3	❐ très souvent	3	❐ jamais
2	❐ assez souvent	2	❐ rarement
1	❐ occasionnellement	1	❐ assez souvent
0	❐ très occasionnellement	0	❐ la plupart du temps
	Je peux rester tranquillement assis à ne rien faire et		J'ai l'impression de fonctionner au ralenti :
	me sentir décontracté :	3	❐ presque toujours
0	❐ oui, quoi qu'il arrive	2	❐ très souvent
1	❐ oui, en général	1	❐ parfois
2	❐ rarement	0	❐ jamais
3	❐ jamais		
	J'éprouve des sensations de peur et j'ai l'estomac		Je ne m'intéresse plus à mon apparence :
	noué :	3	❐ plus du tout
0	❐ jamais	2	❐ je n'y accorde pas autant d'attention que je le devrais
1	❐ parfois		❐ il se peut que je n'y fasse plus autant attention
2	❐ assez souvent	1	❐ j'y prête autant d'attention que par le passé
3	❐ très souvent		
		0	
	J'ai la bougeotte et n'arrive pas à tenir en place :		Je me réjouis d'avance à l'idée de faire certaines choses :
	❐ oui, c'est tout à fait le cas		❐ autant qu'auparavant
3	❐ un peu	0	❐ un peu moins qu'avant
2	❐ pas tellement	1	❐ bien moins qu'avant
1	❐ pas du tout	2	❐ presque jamais
0		3	
	J'éprouve des sensations soudaines de panique :		Je peux prendre plaisir à un bon livre ou à une bonne
	❐ vraiment très souvent		émission radio ou de télévision :
3	❐ assez souvent	0	❐ souvent
2	❐ pas très souvent	1	❐ parfois
1	❐ jamais	2	❐ rarement
0		3	❐ très rarement
	☜ **Total du score pour l'anxiété**		☜ **Total du score pour la dépression**

Chaque réponse correspond à un chiffre. En additionnant ces chiffres, on obtient un score total par colonne (anxiété et dépression). Si le score d'une colonne est supérieur ou égal à 11, cela signifie que vous souffrez d'anxiété ou de dépression (selon la colonne concernée).

Zigmond A.S., Snaith R.P. The Hospital Anxiety and Depression Scale. Acta Psychiatr. Scand., 1983, 67, 361-370

ANNEXE 4. GRILLE AGGIR

Evaluation de la dépendance

Pour la personne âgée concernée, donnez une évaluation : A, B ou C.

- A signifie : Fait spontanément seul, en totalité
- B signifie : Fait partiellement, irrégulièrement, incorrectement, ou sur incitation
- C signifie : Ne fait pas, ne peut pas, refuse de faire

	Variables discriminantes *	A (bon)	B (partiel)	C (non)
**Cohérence**	Converser et se comporter de façon sensée par rapport aux normes admises	○	○	○
**Orientation**	Se repérer dans le temps (jour et nuit, matin et soir), dans les lieux habituels …	○	○	○
**Toilette**	Faire seul, en entier, habituellement et correctement:<br>• la toilette du haut (visage, tronc, membres supérieurs, rasage, coiffage)<br>• la toilette du bas (régions intimes, membres inférieurs, pieds)<br>AA = A CC = C Autres = B	○	○	○
**Habillage**	S'habiller seul, totalement et correctement :<br>• du haut (bras, tête)<br>• du milieu (boutons, ceinture, bretelles..)<br>• du bas (pantalon, chaussettes, bas, chaussures)<br>AAA = A CCC = C Autres = B	○	○	○
**Alimentation**	S'alimenter seul et correctement :<br>• couper la viande, ouvrir un pot, se verser à boire, peler un fruit…<br>• manger seul<br>AA = A CC = C BC = C<br>CB = C Autres = B	○	○	○
**Élimination**	Assure seul et correctement l'hygiène de l'élimination :<br>• élimination urinaire<br>• élimination anale<br>AA = A CC = C AC ou CA = C<br>BC ou CB = C Autres = B	○	○	○
**Transferts**	Se lève (du lit, du canapé, du sol), se couche et s'assoit seul	○	○	○
**Déplacements intérieurs**	Se déplace seul (éventuellement avec canne, déambulateur ou fauteuil roulant)	○	○	○
**Déplacements extérieurs**	Dépasse seul le seuil de sa porte		ne modifie pas le GIR	
**Communication à distance**	Utilise les moyens de communication à distance (cris, téléphone, alarme..)		ne modifie pas le GIR	
	**GIR**			Annuler

D'après un extrait de l'ouvrage *Gériatrie, pour le praticien*, chapitre 110 Evaluation de la dépendance, Philippe Chassagne (55)

Les 7 Variables illustratives suivantes sont non prises en compte dans le GIR :

- Gérer ses propres affaires, son budget et ses biens
- Préparer ses repas et les conditionner pour être servis
- Effectuer l'ensemble des travaux ménagers
- Utiliser un moyen de transport (individuel ou collectif)
- Acquisitions directes ou par correspondance (nourriture, vêtements, objets)
- Se conformer à l'ordonnance du médecin
- Mesurer la socialisation (vacances, animations collectives/organisées…)

ANNEXE 5. SCORE DE CHARLSON : COMORBIDITÉS

I Tableau 6 I

Score de Charlson et adaptation de Quan

Maladie	Pondération initiale [38] (Charlson, 1987)	Adaptation [44] (Quan, 2010)
Infarctus du myocarde	1	0
Insuffisance cardiaque	1	2
Maladie vasculaire	1	0
Maladie neuro vasculaire	1	0
Démence	1	2
Bronchopneumopathie chronique obstructive	1	1
Pathologie rhumatismale	1	1
Ulcère gastro-duodénal	1	0
Hépatopathie peu sévère	1	2
Diabète sans complications	1	0
Diabète avec complications	2	1
Hémiplégie, paraplégie	2	2
Néphropathie	2	1
Cancer (y compris lymphome ou leucémie)	2	2
Hépatopathie modérée à sévère	3	4
Tumeur maligne métastatique	6	6
VIH	6	4
**Score maximal**	**29**	**24**

ANNEXE 6. ORDONNANCE DE PRELEVEMENT BIOLOGIQUE SPECIFIQUE A L'ETUDE

ORDONNANCE POUR L'ESSAI CLINIQUE

GER-SP

Coller étiquette patient ou compléter les données suivantes :

NOM : ……………………………………..

Prénom : …………………………………..

Date de naissance : /_/_/-/_/_/-/_/_//_/_/ (jj-mm-

Contre-étiquette à apposer sur l'étiquette patient : Procédure de pseudonymisation pour enregistrement dans GLIMS

Nom = GERSP- |__|-|__| *(Initiales Nom-Prénom)*

Prénom = |__|-|__||__||__| *(N°centre-N°inclusion)*

DDN : |__|__| / |__|__|__|__| *(mm/aaaa)*

Date de prélèvement :

|__|__| / |__|__| / |__|__|__|__|

Prescription :

☐ Recherche de BNMR sur écouvillon rectal

Nom du prescripteur : I________________________________I

Date : |__|__|/|__|__| / |__|__|__|__| (jj/mm/aaaa)

Signature

PS : Merci de bien vouloir envoyer les résultats à la PARC (coordonnées : service Plateforme d'Appui à la Recherche Clinique, Hôpital de Mercy ; CHR Metz-Thionville, 1 allée du Château - CS 45001 - 57 085 Metz Cedex 03 ; Tél : 03 87 17 98 82 ; courriel : recherche-investigation@chr-metz-thionville.fr)

ANNEXE 7. MODELE D'ETIQUETTES APPOSEES SUR ECOUVILLONS RECTAUX (ESWAB ROSE)

Nom = GERSP- |___|-|___| *(Initiales Nom-Prénom)*

Prénom = |___|-|___||___||___| *(N°centre-N°inclusion)*

DDN : |__|__| / |__|__|__|__| *(mm/aaaa)*

Date de prélèvement :

|___|___| / |___|___| / |___|___|___|___|

ANNEXE 8. MODELE D'ETIQUETTES APPOSEES SUR PRELEVEMENTS EBLSE POSITIFS (CRYOBILLES ET TUBES DE GELOSE)

Type de prélèvement : ___________________________

Référence Patient : |___|-|___||___||___| (*N°centre-N°inclusion)*

Initiales : |___|-|___| *(Initiales Nom-Prénom)*

DDN : |__|__| / |__|__|__|__| *(mm/aaaa)*

Date de prélèvement :

|___|___| / |___|___| / |___|___|___|___|

ANNEXE 9. ECHELLE DE LIKERT

Satisfaction du patient (échelle de Likert) de 0 (non satisfait) à 10 (très satisfait) : |__|__|

ANNEXE 10. AFFICHE PATIENT

Centre Hospitalier Régional
METZ-THIONVILLE

MESSAGE ADRESSÉ AUX PATIENTS ADMIS EN SERVICE DE GÉRIATRIE

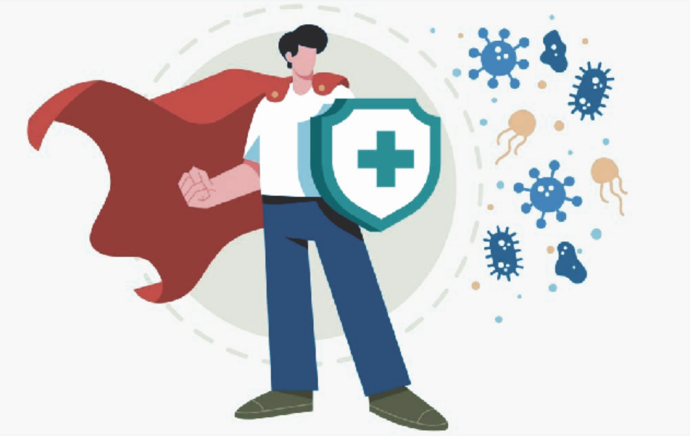

Dans le cadre d'une recherche clinique intitulée :

Impact de l'abandon des précautions complémentaires contact au profit des précautions standard sur la transmission des entérobactéries productrices de β-lactamases à spectre étendu en gériatrie aigue

Le service de gériatrie dans lequel vous êtes hospitalisé, ou votre proche
change ses pratiques d'hygiène !

En effet, dans le cadre de la prévention de la transmission de germes d'un patient à l'autre, nous pensons alléger les précautions actuellement préconisées.

C'est pourquoi nous mettons l'accent sur la promotion des **précautions « standard » d'hygiène** comme par exemple la désinfection régulière des mains ou la désinfection du matériel utilisé pour les patients.

L'étude nous permettra de mieux comprendre les mesures efficaces pour lutter contre les infections nosocomiales.

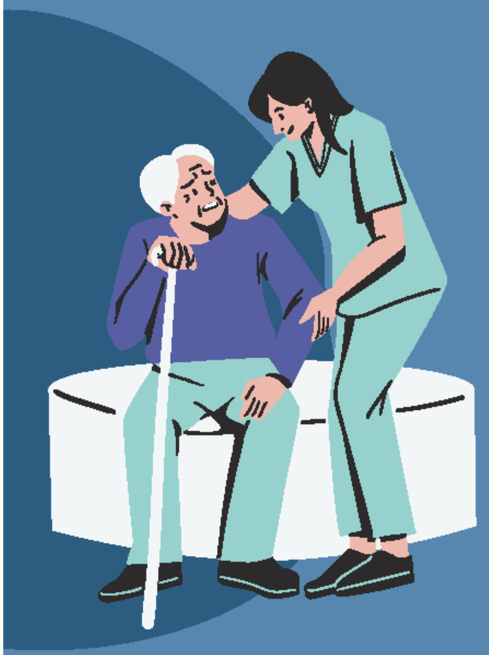

N'HESITEZ PAS A EN PARLER
A L'EQUIPE DU SERVICE
OU PAR TELEPHONE AU
